# H3K23me2 is a new heterochromatic mark in *Caenorhabditis elegans*

**DOI:** 10.1093/nar/gkv1063

**Published:** 2015-10-17

**Authors:** Julien Vandamme, Simone Sidoli, Luca Mariani, Carsten Friis, Jesper Christensen, Kristian Helin, Ole N. Jensen, Anna Elisabetta Salcini

**Affiliations:** 1 Biotech Research & Innovation Centre (BRIC), University of Copenhagen, Ole Maaløes Vej 5, 2200 Copenhagen N, Denmark; 2 Centre for Epigenetics, University of Copenhagen, Ole Maaløes Vej 5, 2200 Copenhagen N, Denmark; 3 Centre for Epigenetics, Department of Biochemistry and Molecular Biology, University of Southern Denmark, 5230 Odense M, Denmark; 4 VILLUM Center for Bioanalytical Sciences, University of Southern Denmark, 5230 Odense M, Denmark; 5 The Danish Stem Cell Centre (Danstem), University of Copenhagen, Blegdamsvej 3, 2200 Copenhagen, Denmark

## Abstract

Genome-wide analyses in *Caenorhabditis elegans* show that post-translational modifications (PTMs) of histones are evolutionary conserved and distributed along functionally distinct genomic domains. However, a global profile of PTMs and their co-occurrence on the same histone tail has not been described in this organism. We used mass spectrometry based middle-down proteomics to analyze histone H3 N-terminal tails from *C. elegans* embryos for the presence, the relative abundance and the potential cross-talk of co-existing PTMs. This analysis highlighted that the lysine 23 of histone H3 (H3K23) is extensively modified by methylation and that tri-methylated H3K9 (H3K9me3) is exclusively detected on histone tails with di-methylated H3K23 (H3K23me2). Chromatin immunoprecipitation approaches revealed a positive correlation between H3K23me2 and repressive marks. By immunofluorescence analyses, H3K23me2 appears differentially regulated in germ and somatic cells, in part by the action of the histone demethylase JMJD-1.2. H3K23me2 is enriched in heterochromatic regions, localizing in H3K9me3 and heterochromatin protein like-1 (HPL-1)-positive foci. Biochemical analyses indicated that HPL-1 binds to H3K23me2 and interacts with a conserved CoREST repressive complex. Thus, our study suggests that H3K23me2 defines repressive domains and contributes to organizing the genome in distinct heterochromatic regions during embryogenesis.

## INTRODUCTION

Chromatin structure is dynamically regulated by reversible covalent post-translational modifications (PTMs), mainly occurring on the N-terminal tails of histone proteins that protrude from nucleosomes. Histones and their reversible PTMs are critical for the dynamic organization of chromatin in structurally and functionally different domains (heterochromatin and euchromatin) and in the regulation of gene expression via the recruitment of chromatin-remodeling enzymes (1). To date, studies have analyzed PTMs of histones in different species, providing evidence that H3 is more extensively modified as compared to the other histone types (2–5), and that the relative abundance and genome localization of different modified histone proteoforms vary among different model organisms (6,7). In the nematode *Caenorhabditis elegans* histone proteins and their PTMs are highly conserved. For example, euchromatic regions contain specific features such as H3K4me3 at the promoters and H3K36me3 in the gene body of active genes, while heterochromatic regions are enriched in methylated H3K9, mainly located on the distal portions of the chromosomes (also called chromosomal arms) (7–9). Heterochromatic regions are also characterized by repetitive elements (10) and by the presence of Heterochromatin Proteins-like 1 (HP1) (9,11,12). The *C. elegans* genome encodes for 2 HP1-like proteins (HPL-1 and HPL-2) localized in discrete foci at the periphery of the nuclei. The distinct sub-nuclear distribution of HP1-like proteins suggests the co-existence of heterochromatic regions with diverse functions (12–14), as also supported by genetic analyses (12). The genome-wide distribution of conserved histone modifications in *C. elegans* has been analyzed by chromatin immunoprecipitation (ChIP), using antibodies raised against some of the best characterized histone PTMs such as the tri-methylated forms of lysine residues 4, 9 and 27 of histone H3 (7,15). However, the nature of this technology precludes the identification of new histone marks and the detection of combinatorial PTMs (16), required to structurally and functionally define sub-nuclear chromatin regions. Indeed, several studies highlight the important role of PTM cross-talk in transcription and epigenetic regulation of the genome (17–22).

We applied an unbiased mass spectrometry (MS) based middle-down proteomics approach (23) to study the variety of histone marks and the relative abundance of co-existing PTMs on H3 tails obtained from *C. elegans* embryos. Similar to mammals, lysine 27 (K27) and lysine 14 (K14) are among the most extensively modified residues. A high degree of methylation is also observed at lysine 23 (K23), which is in contrast to what has been found in mammalian cells (23). Immunofluorescence (IF) microscopy analyses of *C. elegans* embryos show that while acetylation, mono- and tri-methylation of K23 (H3K23ac/me1/me3) are present at similar level in all embryonic cells, di-methylation of K23 (H3K23me2) is reduced in germline precursor cells, in comparison to somatic cells. MS, IF and ChIP approaches indicate that H3K23me2 co-localizes with H3K9me3. IF experiments also highlight the sub-nuclear distribution of H3K23me2 in foci enriched by HPL-1 but not by HPL-2 and, accordingly, *in vitro* biochemical analyses show that HPL-1, but not HPL-2, binds to H3K23me2. Finally, MS analysis of HPL-1 binding partners reveals the specific interaction of HPL-1 with a CoREST-like repressive complex. All together, our findings strongly suggest that H3K23me2, H3K9me3 and HPL-1 define specific sub-heterochromatic regions with gene repression activities.

## MATERIALS AND METHODS

### Genetics and strains

*C. elegans* strains were cultured using standard methods (24). All strains were grown at 20°C. Strains used were as follows: wild type Bristol, *jmjd-1.2(tm3713)*, MT15062: *hpl-1(n4317);hpl-2(tm1489)*, MT17463: *set-25(n5021)*, MT13293: *met-2(n4256), rcor-1(tm4586)*, PFR7(*hpl-1::GFP*) (25) and FR364(*hpl-2::GFP*) (25). *set-25(n5021);met-2(n4256), hpl-1(n4317);rcor-1(tm4586)* and *hpl-2(tm1489);rcor-1(tm4586)* were generated by crossing.

### Microscopy

Fluorescence microscope and DIC pictures were acquired using an automated fluorescence microscope Zeiss (AXIO Imager M2) and MicroManager software, or DeltaVision system and softWoRx software. All pictures were exported in preparation for printing using Fiji (ImageJ).

### Immunofluorescence

For immunostaining, dissected hermaphrodite germlines and embryos were fixed and permeabilized as described (26). Polyclonal antibodies raised against: H3K23ac (07–355, Millipore), H3K23me1 (39387, Active Motif), H3K23me2 (39653, Active Motif), H3K23me3 (61499, Active Motif) and monoclonal raised against: H3K4me2 (05–1338, Millipore), H3K9me2 (ab1220, Abcam), H3K9me3 (39286, Active Motif), H3K27me3 (ab6002, Abcam), PGL-1 (OIC1D4, DSHB) and GFP (11814460001, Roche) were used. Secondary antibodies were: goat anti-mouse IgG (Alexafluor 488), goat anti-rabbit IgG (Alexafluor 594), both purchased from Invitrogen. Mounting medium for fluorescence with DAPI (Vectashield H1200) was used to counterstain DNA. Experiments were performed in triplicate and more than 30 germlines and 50 eggs were scored in total. All antibodies were diluted 1:100–1:500 in PBS-T.

### GFP pull-down

Total protein extracts from animals carrying the *hpl-1::GFP* transgene were obtained by grinding a frozen pellet of mixed eggs and L1 with a mortar and pestle into powder, and resuspended in IP buffer A (150 mM KCl, 0.1% Igepal, 1 mM EDTA, 1 mM MgCl_2_, 10% glycerol, 50 mM Tris HCl [pH 7.4] and protease inhibitors). After centrifugation, the supernatant was saved as extract A and the pellet was resuspended in IP buffer B (300 mM KCl, 0.2% Igepal, 1 mM EDTA, 1 mM MgCl_2_, 10% glycerol, 50 mM Tris HCl [pH 7.4] and protease inhibitors). Magnetic GFP-Trap beads (Chromotek) were used to precipitate GFP-tagged proteins from lysates and blocked magnetic beads for pre-clearing. Approximately 200 mg of total proteins were used for the pull-down. Following incubation and washes with the same buffers, proteins were eluted with acidic glycine (0.1 M [pH 2.5]), resolved on a 4–12% NuPage Novex gel (Invitrogen), and stained with Imperial Protein Stain (Thermo Scientific). The gel was sliced into five bands across the entire separation range of the lanes. Cut bands were reduced, alkylated with iodoacetamide, and in-gel digested with trypsin (Promega) as previously described (27), prior to LC/MS-MS analysis.

### Mass spectrometry of proteins eluted from HPL-1::GPF IP

Equipment and set-up, as well as database search were performed as previously described (28).

### Protein interaction assay

For co-immunoprecipitation assays, frozen eggs from transgenic lines carrying *hpl-1::GFP* and *hpl-2::GFP* (prepared by hypochlorite treatment) were reduced into powder using a mortar and pestle. The powder was resuspended in IP buffer B (described in GFP pull-down section) and 2 mg of total proteins were incubated with blocked magnetic particles beads (bmp, Chromotek) overnight at 4°C. Pre-cleared soluble fractions were collected and incubated with Magnetic GFP-Trap beads (Chromotek) for 2 h at 4°C. Beads were washed five times in IP buffer B, boiled in SDS-sample buffer and analyzed by SDS-PAGE followed by western blotting. Antibodies used in this experiment were: anti-SPR-5 (sc-68340, Santa Cruz Biotechnology) at 1:100, anti-GFP (Roche, 11814460001) at 1:5000 and peroxidase-labeled anti-rabbit and anti-mouse secondary antibodies (Vector) at 1:10 000.

### Nuclear extraction, histones purification and digestion prior to LC-MS/MS analysis

Two samples of 2 ml of packed embryos from wild-type animals were collected by hypochlorite treatment. Nuclear extract was performed as previously described (8), without fixation. Purified nuclei were resuspended in Nuclear Lysis Buffer (50 mM Tris [pH 7.4], 1.5 mM MgCl_2_, 420 mM NaCl, 1 mM DTT, 2 mM EDTA, 20% glycerol and protease inhibitors) and sonicated 10 cycles of 30 s ON–30 s OFF, HIGH power (Bioruptor). Histones were purified following the described protocol (29) with minor modifications. Briefly, nuclear extracts were resuspended in 0.2 M H_2_SO_4_ and shacked for 45 min at room temperature. The histones present in the supernatant were precipitated with 33% trichloroacetic acid (TCA) for 1 h. Protein abundance was estimated as ∼20–30 μg. Digestion was performed overnight at room temperature in 50 mM NH_4_CO_3_ (pH 8.0) by using protease GluC (Calbiochem) with an enzyme:sample ratio of 1:50.

### Liquid chromatography

Histone tails were separated as previously described (23) using a Dionex Ultimate 3000 nanoLC (Thermo Scientific) controlled by Chromeleon software. For each of the two biological replicates, two technical replicates were run. The nanoLC was equipped with a two column setup, a 5 cm pre-column (100 μm ID) packed with C_18_ bulk material (ReproSil, Pur C18AQ 5 μm; Dr Maisch) and a 22 cm analytical column (75 μm ID) with picofrit packed with Polycat A resin (PolyLC, Columbia, MD, 3 μm particles, 1500 Å). Loading buffer was 0.1% formic acid in water. Buffer A and B were prepared as described previously (30). Briefly, solvent A was 75% acetonitrile, 20 mM propionic acid, adjusted to pH 6.0 using ammonium hydroxide, and solvent B was 25% acetonitrile adjusted to pH 2.5 with formic acid. The column oven of the nanoLC was set to 30°, in order to decrease trap pressure. Sample was loaded onto the pre-column for 10 min with loading buffer at 3 μl/min. Samples were run with a gradient of 5 min 100% solvent A, followed by 55–85% solvent B in 135 min and 85–100% in 10 min for column washing. Flowrate for the analysis was set to 250 nl/min.

### Tandem mass spectrometry

The nanoLC was coupled on-line with an LTQ-Orbitrap Velos (Thermo Scientific, Bremen, Germany) equipped with an ETD source for fragmentation. Nanoelectrospray (Proxeon, Odense, Denmark) was used with a spray voltage of 2.2 kV. No sheath, sweep and auxiliary gasses were used, and the capillary temperature was set to 270°C. Dynamic exclusion was disabled during data acquisition. Data acquisition was performed in the Orbitrap for both precursor ions and product ions, with a resolution of 60 000 (full-width at half-height) for MS and 30 000 for MS/MS. Precursor charge states 1+, 2+ and 3+ were excluded. Isolation width was set at 2 *m/z*. The six most intense ions were isolated for fragmentation using ETD with an activation Q value of 0.25, activation time of 90 ms and supplementary activation. Charge state dependent ETD reaction time was enabled. MS/MS acquisition was set with 3 microscans, an automatic gain control (AGC) of 2 × 10e5 and a maximum injection time of 800 ms. Acquisition window was set at *m/z* 450–750, which included histone H3 tail charge states from 7+ to 11+.

### Data processing and analysis

Spectra deconvolution was performed with Xtract with the following parameters: S/N threshold 0, resolution at 400 *m/z* 30 000 and monoisotopic mass only TRUE. Raw files were processed and searched using Proteome Discoverer 1.4.0.288. Mascot (Matrix Science, London, UK) was chosen as database searching engine. Search parameters were as follows: MS mass tolerance: 2.2 Da, to include possible errors in isotopic recognition. MS/MS mass tolerance: 0.01 Da. Enzyme: GluC, no missed cleavages. The database sequence was manually curated in order to include all the *C. elegans* histone isotypes (downloaded from Uniprot 12/2012). Variable modifications were mono- and di-methylation (K, R), tri-methylation (K) and acetylation (K). XML result file from Mascot was imported and processed in the in-house developed 'Histone Coder′ by using a tolerance of 30 ppm. Histone Coder annotated the number of site determining fragment ions for each assigned PTM, and only PTMs with at least one site, determining ion in both directions of the sequence were accepted. Quantification was performed by using the in-house software isoScale, which extracts the total ion intensity from MS/MS spectra. The total ion intensity was summed for all peptide–spectrum matches and normalized by the sum of all histone H3 N-terminal tails quantified (23). In case of mixed spectra isoScale calculated the fragment ion relative ratio (FIRR) of two isobaric peptides contained in the same MS/MS spectrum and divided the total ion intensity to the two species. The MS/MS tolerance to identify fragment ion for FIRR calculation was set to 0.05 Da. Histone Coder and isoScale are available at http://middle-down.github.io/Software/. The interplay value was calculated according to our previous publication (21) as follows: interplay value = log_2_((co-existence frequency mark XY) / ((co-existence frequency mark X)(co-existence frequency mark Y))).

### Recombinant protein production and peptide pull-down assays

The full-length sequences of *hpl-1* and *hpl-2a* were cloned in pGEX-KT (Addgene). HPL-1 and HPL-2a GST fusion proteins were expressed in *E. coli* BL21 (DE3) and purified on glutathione-Sepharose 4B (GE Healthcare) according to the manufacturer's instructions. GST-proteins were eluted with 10 mM glutathione in 50 mM Tris-HCl (pH 8.0). The purity of each recombinant protein was tested after migration on polyacrylamide gel and staining with SimplyBlue SafeStain (Invitrogen). Synthetic biotinylated peptides corresponding to histone H3 tails with/without modification were generated by JPT Peptide Technologies: 11–30 K23me0, 11–30 K23me1, 11–30 K23me2, 11–30 K23me3, 1–44 K9me3, 1–44 K27me3. Peptide pull-downs were performed as previously described (31). Each biotinylated peptide was coupled to streptavidin-dynabeads (Invitrogen). 1 μg of recombinant protein: GST, HPL-1::GST and HPL-2a::GST, diluted in PD 150 buffer (20 mM Hepes [pH 7.9], 150 mM KCl, 0.2% Triton-X 100, complete protease inhibitor cocktail, 20% glycerol), was incubated for 2 h at 4°C with the beads. Beads were washed six times with PD 150 buffer. Bound proteins were separated on polyacrylamide gel, followed by a western blot analysis with GST antibody (27–4577–01, Amersham).

### Purification of JMJD-1.2 and demethylase assays

Purification of Flag-tagged recombinant JMJD-1.2 from insect cells and demethylase assays were performed as previously described (32) using *C. elegans* histones (acid extracted from nuclear extracts) as substrates. Reaction mixtures were analyzed by western blotting using specific antibodies.

### Construction and analysis of *hpl-1;rcor-1* and *hpl-2;rcor-1* double mutants

*hpl-1(n4317);hpl-2(tm1489)* or *hpl-2(tm1489)* hermaphrodites were crossed with males *rcor-1(tm4586)* at 15°C using standard crossing conditions. Several lines *hpl-1(M+, Z-);rcor-1* and *hpl-2(M+, Z-);rcor-1* were recovered and their progeny (which have lost maternal contributed HPL-1 or HPL-2) analyzed for vulva defects and sterility, under dissecting microscope. Lines were defined vulva defective when at least 5% of animals in the plate showed aberrant vulva (protruding vulva or multivulva) and lines were defined sterile when failed to produce any viable progeny. The analysis of *hpl-2;rcor-1* vulva defects was done in fertile lines and performed at 15°C and 20°C as this double mutant shows fully penetrant sterility at 25°C.

### Chromatin immunoprecipitation from embryos

To perform ChIP from wild-type embryos, we followed the protocol described (33), using rabbit IgG (Sigma) and H3K23me2 (39653, Active Motif) as antibodies. The ChIP experiments were performed in duplicate and the DNA sequenced by The Danish National High-Throughput DNA Sequencing Centre.

### Origin and processing of NGS ChIP-seq data

Publicly available ChIP-seq data were obtained from the modEncode website (10,34,35) for the following histone marks: H3K9me3, H3K27me3, H3K23ac, H3K27ac, H3K36me2 and H3K36me3 (modEncode IDs 5153, 5163, 5151, 5159, 5164 and 5165). All data sets were available in duplicates, and in all cases, the raw reads were downloaded. All raw reads were aligned against the ce10 reference genome using bowtie2 (36). The resulting alignments were then processed using the spp package for R (R Core Team, 2014. R: A language and environment for statistical computing. R Foundation for Statistical Computing, Vienna, Austria), according to the authors suggestions (37). All sequence tags were filtered using the functions binding.characteristics and select.informative.tags to select informative tags based on the binding characteristics. Next, singular positions with very high tag counts (specify) were filtered using the remove.local.tag.anomalies function (37). This approach scans along the chromosomes, calculating local density and removing or restricting singular positions with extremely high tag count relative to the neighborhood (37). Similar process was applied to the H3K23me2 ChIP-seq data.

### Genome-wide tag density correlations

Genome-wide smoothed tag density profiles for each histone mark were derived using the get.smoothed.tag.density function from package spp with the IgG wild type sample as background. From these profiles a traditional Pearson correlation was calculated both at the individual replicate level and using a data set in which the individual bowtie alignments from each replicates were merged. In all cases but one, the correlation between duplicates was above 0.9, which was taken as justification for using the merged data. The exception was for H3K23ac, where the two duplicates showed a correlation of 0.78. We selected the first replicate (ID 5151) as, in comparison to the second, it showed the expected enrichment at promoters of transcribed genes, as described in Ho *et al*., 2014 (7). Figure 5A was drawn on the merged data using the corrplot package for R (Wei, T., 2013. corrplot: Visualization of a correlation matrix. R package version 0.73 ed.).

### Chromosomal tag enrichment/depletion profiles

Enrichment/depletion profiles were calculated using the get.conservative.fold.enrichment.profile function, also from spp, which scans the ChIP-seq data and signal tag density to estimate significant lower bounds of tag enrichment and upper bound of tag depletion. A significance level (alpha) of 0.01 was used. Figure 5B was drawn using the ggplot2 (38) package for R.

## RESULTS

### Lysine residues 14, 23 and 27 of histone H3 are heavily modified by methylation and acetylation in *C. elegans* embryos

**Figure 1.**
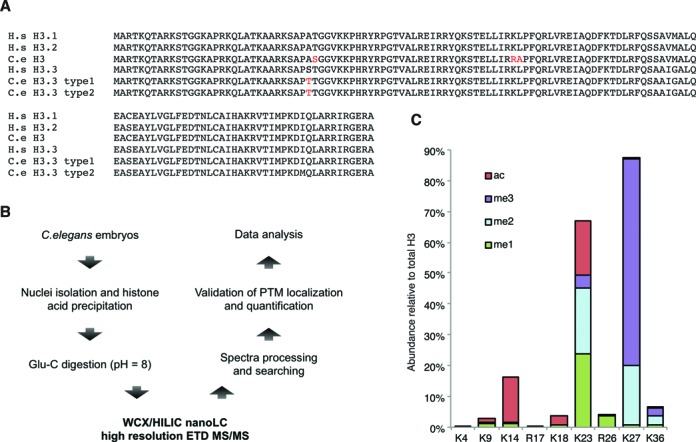
Post-translational modifications identified on histone H3 N-terminal tail (1–50) from embryos, by middle-down analysis. (**A**) Alignment of histone H3 sequences from *Homo sapiens* (H.s) and *Caenorhabditis elegans* (C.e). Differences in amino acid sequences between species are highlighted in red. (**B**) Schematic representation of the middle-down proteomics workflow. Histones were purified from worm embryos by cell nuclei isolation followed by acid extraction; histones were then digested with Glu-C to obtain the 50 aa N-terminal peptides; sample was analyzed by LC-MS/MS; tandem mass spectra were used as input for the Mascot sequence database search engine and results were filtered and quantified with in-house software. (**C**) Relative abundance of most abundant histone H3 PTMs. Color codes are: red, acetyl; green, mono-methyl; pale blue, di-methyl; violet, tri-methyl.

Histone proteins are highly conserved throughout the evolution of eukaryotes. The alignment of histone H3 amino acid sequences from *Homo sapiens* and *Caenorhabditis elegans* revealed only a few amino acid sequence mismatches between the different histone isoforms of the two organisms (Figure 1A), indicating that H3 is a highly conserved component of chromatin in eukaryote species. We initially investigated whether *C. elegans* histone proteins, specifically H3, are post-translationally modified to the same extent as for other organisms. We used our optimized middle-down proteomics workflow, consisting of a nanoliter flow hybrid reversed-phase/hydrophilic interaction liquid chromatography coupled to electrospray ionization tandem mass spectrometry (RP/HILIC-MS/MS) platform (23). Histone N-terminal tails were sequenced for global mapping of the combinatorial histone marks, using two biological replicates of *C. elegans* embryos as starting material (Figure 1B). The good reproducibility across biological and technical replicates indicates that the strategy adopted was reproducible (Supplementary Table S1, average R^2^ correlation = 0.94). The relative abundance of PTMs on the histone tails was determined by summing the ion abundances of all peptide species containing a particular modification, and dividing by the total number of peptides identified, as previously reported (23) (Figure 1C). We quantified a total of 32 different marks at 12 distinct amino acid residues on the histone H3 tails (aa 1-50) (Supplementary Figure S1A). H3K27, H3K14 and H3K23 were the most extensively modified residues (Figure 1C and Supplementary Figure S1A). Notably, methylated forms of H3K23 occupy almost 50% of all histone H3 in *C. elegans* embryos. All other histone PTMs identified in *C. elegans* embryos were below 5% of total PTM occupancy, including methylated forms of H3K9 and H3K36. Specifically, methylations on H3K9 were less than 2% (1.2% me1, 0.36% me2 and 0.27% me3) in *C. elegans* embryos, in contrast to their high abundance detected in mouse embryonic stem cells (mESC) (>50%) as determined by using an identical proteomics approach (23). Methylation of H3K4 was also barely detected with our approach. However, this is expected, as K4 methylation has been always reported as one of the lowest abundant methylations on histone H3 (2,39–41). Finally, the abundance of H3K36 modifications differed dramatically between worm and mouse, with less than 10% of H3K36 being modified in *C. elegans* (this study) versus almost 90% in mESC (23).

Thus, the middle-down proteomics approach determined a series of histone modifications of H3, including individual and combinatorial marks, and identified several unique features of *C. elegans* H3 PTMs as compared to current knowledge derived from histone studies in mammals.

### Cross-talk of post-translational modifications on histone H3 tails

**Figure 2.**
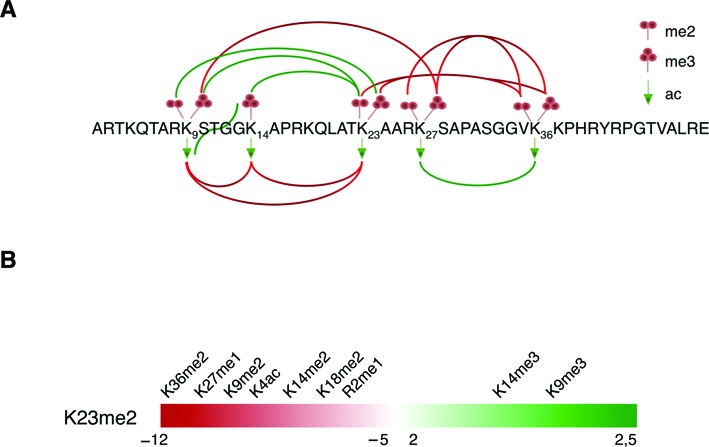
Histone H3 cross-talk. (**A**) Cartoon of histone H3 N-terminal tail (aa 1–50) and the experimentally and computationally determined interplay between lysine di-, tri-methylations and acetylations. Green and red lines represent positive and negative interplays, respectively. For clarity, the image displays only the most pronounced interplay values. (**B**) Interplay values of binary marks from H3K23me2 perspective. Major interplay values are represented as negative score (red) = rarely together on the same tail, positive score (green) = frequently together on the same tail.

Combinations of distinct co-existing histone PTMs influence local chromatin conformation and activity. This is often referred to as PTM cross-talk (42). The majority of the polypeptides identified in this study contained at least two PTMs (∼73%) (Supplementary Figure S1B), whereas only ∼27% contained no or one modification, suggesting that most of the histone H3 tails carry multiple PTMs. The most abundant combinations of binary PTMs were K23me2:K27me3 (19.22%), K23me1:K27me3 (18.49%) and K23ac:K27me3 (12.57%) (Supplementary Table S1). This result is in line with the evidence that H3K27me3 mark is the most abundant modification detected on histones (67.32%). In order to study PTM cross-talk in *C. elegans*, we calculated the co-existence frequency of the detected modifications on histone H3 tails and determined positive and negative interplay scores for pairs of modifications by using our recently described mathematical model (21). Briefly, we compared the observed co-existence frequency of two PTMs to their expected co-existence frequency by calculating the interplay using the following formula: log_2_(observed frequency/expected frequency), where the expected frequency is determined as the product of the relative abundance of the two given PTMs (PTM_1_ x PTM_2_). Positive interplay values correspond to histone marks that co-exist with a higher frequency than expected for random deposition, while negative interplay values indicate lower frequency than expected. Highly positive (score >>0) or negative (score <<0) interplays suggest a remarkable PTM interdependency, and thus cross-talk. We extracted 328 combinations of binary histone H3 marks with a negative interplay value and 132 binary marks with a positive interplay value (Supplementary Table S1). This suggests that functional positive interplay is limited to rather few distinct combinations of histone marks. Many more candidate combinations of binary marks exhibit a mutually exclusive behavior rather than co-dependency (Supplementary Figure S1C). Among the combinations of binary marks with the most negative values (score <−10) we identified K36me1/2/3 with K27me2/3, indicating, as expected, that the methylation of K36, which is frequently associated with actively transcribed genes, is not common on histone tails carrying the repressive marks K27me2/3. Of note, we did not observe any peptide containing both PTMs K27me2 and K36me2 (Supplementary Table S1), which appeared surprising as this is one of the most abundant binary marks in mouse embryonic stem cells (23), however, this result is in line with the low levels of K36me2 in *C. elegans* as compared to mESC. We also found that K9me2/3 is mutually exclusive with K27me2/3, as also reported for other organisms (43,44). Highly negative interplay values were determined also for K36me1/2/3 and K23me2/3. Positive interplay values were observed for K27ac:K36ac, K9me2:K23me3 and K9me3:K23me2, with positive scores of 4.28, 4.32 and 2.21, respectively. A summary of the most positive and negative interplay relationships between pairs of H3 marks is shown schematically in Figure 2A.

Next, we investigated whether the very abundant H3K23me2 mark of *C. elegans* generated significant interplay scores with other H3 marks (Figure 2B and Supplementary Table S1). K23me2 exhibited a positive interplay score with K9me3 and K14me3. Specifically, even though the H3K9me3 mark was overall low abundant, it was found sharing the same histone tail with H3K23me2. Negative interplay scores were found for H3K23me2 with K36me2, K27me1 and K9me2 (Figure 2B). Interestingly, H3K23me3 had opposite behavior with H3K9me2/3, suggesting the existence of a finely regulated cross-talk between K9 and K23 modifications (Figure 2A and Supplementary Table S1).

Thus, our analysis shows that H3K23me2 co-exists on the same histone tail with H3K9me3 and is mutually exclusive with H3K36me2, suggesting that H3K23me2 might be associated with transcriptionally silent chromatin regions.

### H3K23 localization in embryos and germ cells

**Figure 3.**
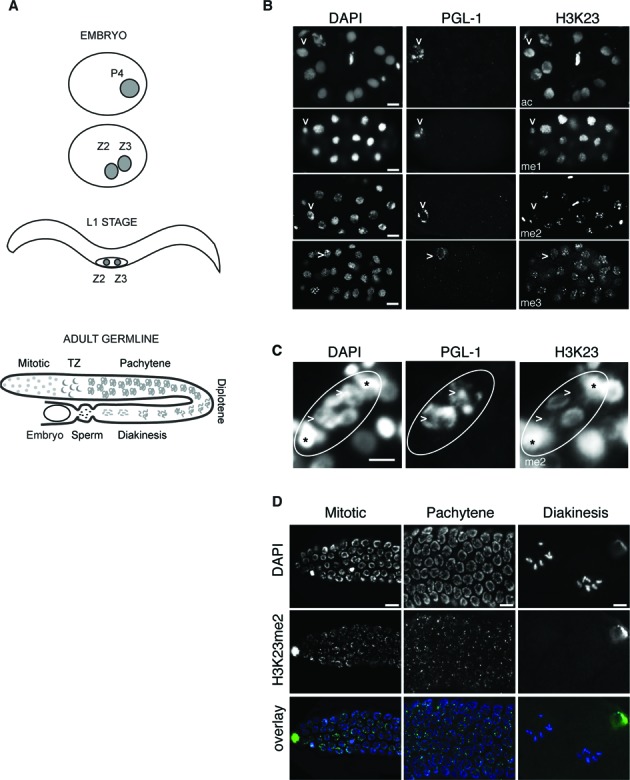
Modulation of H3K23me2 in germ cells. (**A**) Schematic representation of the gonadogenesis in *C. elegans*. The germline lineage is represented in gray. (**B**) Representative images of wild type embryos (n > 50) fixed and co-stained with the indicated antibodies. PGL-1 antibody (middle column) recognizes a specific germ cell protein. DNA is counterstained with DAPI (left column). In P4 blastomeres (indicated with arrowheads) H3K23me2 appears reduced. (**C**) Representative images of Z2 and Z3 in L1 (n > 50) fixed and stained with antibodies against H3K23me2 (right) and PGL-1 (middle). DNA is counterstained with DAPI (left). Arrowheads indicate Z2 and Z3 precursor germ cells, circles indicate the gonad and asterisks indicate putative precursors of the somatic gonad (Z1 and Z4), based on their position. (**D**) Representative images of extracted gonads (n > 30) from adult wild-type animals fixed and stained with H3K23me2 antibody (middle row). DNA is counterstained with DAPI (top). Overlay pictures are presented with artificial colors (bottom). Specific regions of the germline are shown (mitotic zone, pachytene zone and diakinesis). H3K23me2 is not detected in the diakinesis region. Scale bars are 5 μm.

As H3K23 is highly modified in embryos, we further investigated the presence of H3K23 modifications during embryogenesis using IF microscopy analyses. In embryos, both somatic and precursor germ cells are present, with P4 being the germ line founder and first primordial germ cell (PGC). Its symmetric division, at about the 100-cell stage, gives birth to two PGCs called Z2 and Z3. These two cells are arrested in G2 or in early prophase throughout embryogenesis (45,46) and resume proliferation after hatching, during the first larval stage (L1), to form the adult germ line (47) (Figure 3A). The chromatin of PGCs is unique in comparison to the neighboring somatic cells, showing a remarkable condensation (48) and reduced levels of linker histone H1.1, H3K4me2/me3, H3K27me2 and H4K8ac in Z2/Z3 cells (48–51). By IF, PGCs can be distinguished from somatic cells by the presence of PGL-1, a component of the P granules expressed only in germ cells (52). After validation of the specificity of H3K23me antibodies by dot blot and peptide competition assays (Supplementary Figure S2), we performed IF on wild type embryos (Figure 3B). We found that H3K23 modifications are ubiquitously present in embryonic somatic cells but, while H3K23ac and H3K23me1 are broadly diffused in all the nuclei, H3K23me2 and H3K23me3 are localized in bright and discrete areas or foci (Figure 3B). Interestingly, while H3K23me3 is present in PGCs at similar levels compared to somatic cells, H3K23me2 appears reduced in PGCs both in embryos and in freshly hatched larvae (Figure 3B, C and Supplementary Figure S3). These results suggest a specific modulation of H3K23me2 levels in the germ cell precursors.

We also analyzed the presence and the regulation of this mark in fully developed germline. The adult hermaphrodite germline is organized in a U-shaped tubular structure with mitotic cells located at the distal region. Mitotic cells enter meiosis in a so-called transition zone and progressively migrate to the more proximal region of the gonad while maturating to oocytes through pachytene and diakinesis (Figure 3A). In adult germline, H3K23me2 has a dotted appearance and is present both in mitotic and meiotic regions, but is not detected in the oocytes (diakinesis region), where chromosomes shrink and shape into six discrete bivalents (Figure 3D). The specific reduction of H3K23me2 in PGCs and in oocytes indicates a tight regulation of this mark in germ cells and suggests that this modification is reduced when chromosomes are highly condensed.

### The histone demethylase JMJD-1.2 is required for proper regulation of H3K23me2 in adult germline

**Figure 4.**
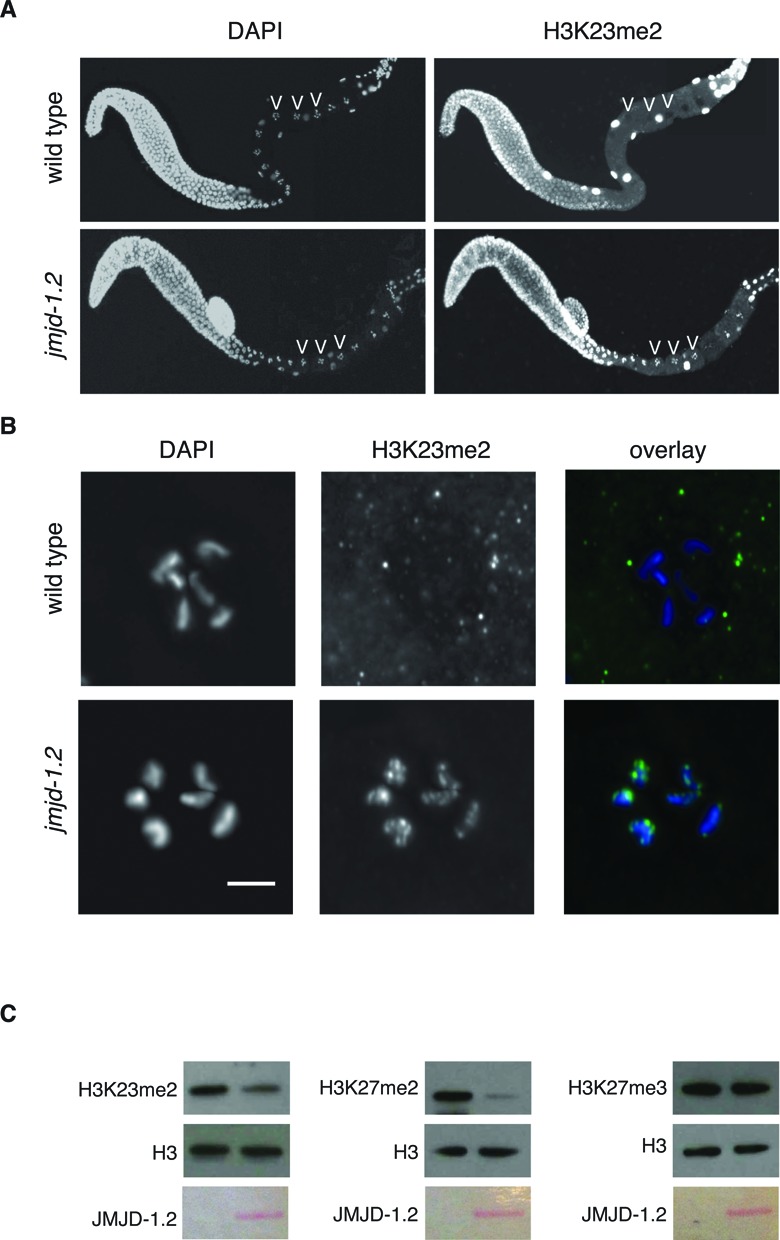
The histone demethylase JMJD-1.2 regulates H3K23me2 levels in the oocytes. (**A**) Extracted gonads from wild type (top) and *jmjd-1.2(tm3713)* (bottom) were fixed and stained with H3K23me2 antibody (right). DNA was counterstained with DAPI (left). Arrowheads indicate some oocytes nuclei at the diakinesis stage. (**B**) Magnification of oocytes from extracted gonads of wild type and *jmjd-1.2(tm3713)* adult hermaphrodite fixed and stained with H3K23me2 antibody (middle). DNA was counterstained with DAPI (left). Overlay (right). Scale bar is 5 μm. (**C**) Demethylation assay on *C. elegans* histones incubated with recombinant JMJD-1.2 (30 μg). The reaction was probed with the indicated antibodies. Ponceau staining of purified JMJD-1.2 used for the reaction is presented at the bottom.

To obtain information regarding enzymes regulating the levels of H3K23me2, we screened available mutants of experimentally tested or putative lysine demethylases (KDMs) and methyltransferases (KMTs), for aberrant modulation of H3K23me2 in PGCs and in adult germline. A list of KMTs and KDMs that we tested is presented in Supplementary Table S2. We assumed that loss of the specific methyltransferase would reduce the level of H3K23me2, while loss of the demethylase would enhance it. We were not able to identify any KMT or KDM modulating the level of H3K23me2 in embryos. In adult germline, however, H3K23me2 appears aberrantly regulated in *jmjd-1.2* mutant. In this mutant strain, the H3K23me2 staining was evident in the diakinesis region, where it is normally undetectable in wild-type worms (Figure 4A and B). We, and others, previously reported that JMJD-1.2 acts as a demethylase for H3K9me2 and H3K27me2 (32,53). To test whether JMJD-1.2 also possesses intrinsic activity toward H3K23me2, we affinity purified the full-length recombinantly expressed protein (Supplementary Figure S4) and tested its activity *in vitro* (Figure 4C). Incubation of the purified protein with *C. elegans* histones shows, as expected, a decrease of H3K27me2 but not H3K27me3. Remarkably, purified JMJD-1.2 was also able to demethylate H3K23me2. Taken together, our results strongly suggest that JMJD-1.2 directly regulates H3K23me2 levels specifically in the adult germline and that the dynamic of this PTM in germ cells is differentially regulated during embryogenesis and the adult life.

### H3K23me2 is enriched in heterochromatic regions

**Figure 5.**
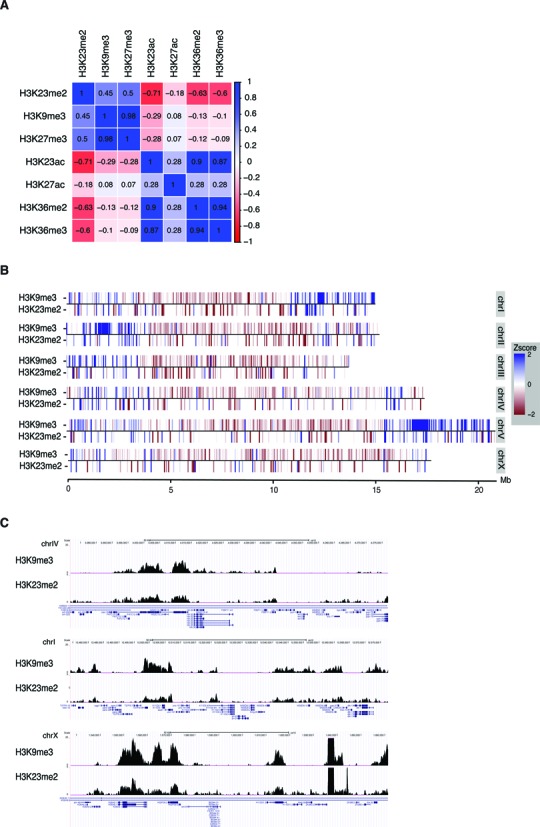
H3K23me2 correlates with heterochromatic marks and is enriched in chromosomal arms. (**A**) Genome-wide correlation matrix of H3K23me2 with H3K9me3, H3K27me3, H3K23ac, H3K27ac, H3K36me2 and H3K36me3. (**B**) Chromosomal heat maps depicting the enrichment/depletion profiles of H2K23me2 and H3K9me3 according to chromosomal location. Red indicates depletion, blue enrichment. (**C**) Genome browser screenshots of background-subtracted signals of H3K23me2 and H3K9me3 in different chromosomes.

To further investigate the genome-wide localization of H3K23me2 we performed a chromatin immunoprecipitation using the H3K23me2 antibody followed by deep sequencing (ChIP-seq). Genome-wide Pearson correlation coefficients for median Z-scores of the ChIP signals between H3K23me2 and other selected marks are shown in Figure 5A. H3K23me2 positively correlates with H3K9me3 and H3K27me3, marks enriched in heterochromatic regions (7,9,15) and negatively correlates with H3K36me2/3 and H3K23/27ac, modifications enriched in actively transcribed regions. We also tested by ChIP–qPCR genomic regions in which distinct peaks of H3K9me3 have been reported by the modENCODE consortium (Supplementary Figure S5). As shown in Supplementary Figure S4, H3K23me2 is enriched in H3K9me3 but not in H3K4me3 positive regions, used as negative control, further confirming the presence of H3K9me3 and H3K23me2 on the same genomic regions.

Distribution patterns along the chromosomes have been reported for several post-translational modifications (7), showing that central chromosomal regions carry a characteristic epigenetic signature distinct from more peripheral regions, also denominated chromosomal arms. In general, compared to the central regions, chromosomal arms are enriched with repressive histone marks, in particular methylation of H3K9, a hallmark of heterochromatin. The X chromosome has a peculiar distribution of the marks (29), with increased presence of H4K20me1 and H3K27me1, possibly related to dosage compensation, and with H3K9me1/2/3 enriched in the left arm (15). We analyzed the distribution of H3K23me2 and chromosomal heat maps depicting median Z-scores of ChIP signals for H3K23me2 are shown in Figure 5B. This analysis highlights the H3K23me2 enrichment (blue lines) on autosomal arms and its depletion (red lines) from the central regions of the autosomes and from most of the length of the X. This distribution pattern is similar to the one reported for H3K9me3 (7,9), as also shown in the genome browser snapshots (Figure 5C), further supporting that H3K23me2 is a mark enriched in heterochromatic regions.

### H3K23me2 co-localizes with H3K9me3 and HPL-1, but its deposition is independent of HPL proteins and H3K9 methylation

**Figure 6.**
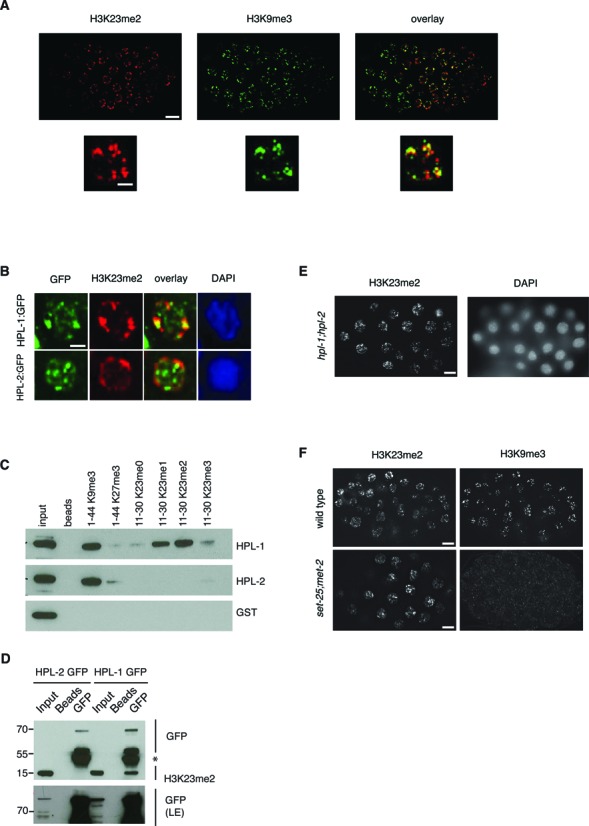
H3K23me2 co-localizes with H3K9me3 and HPL-1, which binds directly H3K23me2. (**A**) Representative images of wild type embryos fixed and stained with H3K23me2 (red) and H3K9me3 (green) antibodies. Overlay is shown (right). Magnifications of representative single nuclei are shown below. (**B**) Embryos from transgenic animals expressing HPL-1::GFP and HPL-2::GFP were fixed and stained with H3K23me2 (red) and GFP (green) antibodies. DNA was counterstained with DAPI (blue). Representative individual nuclei are shown. (**C**) Purified GST-HPL-1 and GST-HPL-2a fusion proteins were incubated with biotinylated histone H3 peptides methylated at K9, K27 or K23 position. After precipitation with Streptavidin-dynabeads, bound GST-HPL proteins were separated in SDS-PAGE and detected by immunoblotting with GST antibody. (**D**) Lysates from transgenic lines carrying GFP-tagged HPL-1 and HPL-2 were immunoprecipitated with GFP antibody, and probed with H3K23me2 and GFP antibodies. Asterisk (*) indicates immunoglobulins reacting with the secondary antibodies. A long exposure (LE) of the GFP immunostaining is presented at the bottom to detect the inputs. (**E**) Representative images of embryos from *hpl-1;hpl-2* double mutant, fixed and stained with H3K23me2 antibody. DNA is counterstained with DAPI. (**F**) Representative images of embryos from wild type (top) and *set-25;met-2* double mutant (bottom), fixed and stained with H3K23me2 (left) and H3K9me3 (right) antibodies. Scale bars: 5 μm for full embryos in **A**, **D** and **F**, 2 μm for single nuclei in **A** and **B**.

By IF, H3K23me2 shows a dotted pattern (Figure 3) similar to the one reported for H3K9me3 during embryogenesis (14,54). We performed co-immunostainings of H3K23me2 with H3K9me3 and found that H3K23me2 partially co-localizes with H3K9me3 (Figure 6A), but not with the other histone PTMs analyzed (Supplementary Figure S4), suggesting that this approach could be used to further characterize the H3K23me2 mark. In mammals, H3K9me3 is found in heterochromatic regions that are bound by the HP1 chromodomain proteins (55). There are two genes coding for HP1 orthologs in *C. elegans*, *hpl-1* and *hpl-2*. The two HP1 proteins are reported to define distinct sub-nuclear regions in embryonic nuclei, based on IF analyses (12,14,56). We took advantage of two transgenic strains expressing HPL-1 and HPL-2 fused to GFP (25) to test the co-localization of the tagged proteins with H3K23me2. As previously reported, tagged HPL-1 and HPL-2 localize in foci (Figure 6B). Co-staining with H3K23me2 shows that tagged HPL-1, but not HPL-2, partially co-localizes with H3K23me2 and H3K9me3 (Figure 6B, Supplementary Figure S6A and S6B). To address whether HPL-1 could directly bind methylated forms of H3K23, we tested the binding capacities of recombinant HPL-1 and HPL-2 fused to GST (Supplementary Figure S6C) on synthetic peptides containing K9me3, K27me3 and K23me0–3, using a peptide pull-down approach. As expected (55), both recombinant proteins bound strongly to the H3K9me3 peptide but not to H3K27me3. Strikingly, HPL-1, but not HPL-2, also bound to methylated H3K23, in particular for the di-methylated version (Figure 6C). In addition, HPL-1, but not HPL-2, has the ability to bind H3K23me2 in immunoprecipitation assays (Figure 6D). These experiments suggest that HPL-1 could be recruited to discrete foci through its direct interaction with H3K23me2.

To investigate whether the formation of H3K23me2 foci depends on HPL proteins and/or H3K9me3, we used genetic approaches. IF staining with H3K23me2 in embryos derived from the double mutant *hpl-1;hpl-2*, lacking both HPL proteins, shows a distribution of H3K23me2 in dotted structures similar to wild-type animals (Figure 6E), indicating that HPL proteins are dispensable for H3K23me2 localization in foci. Also, in absence of all methylated forms of H3K9, as in the case of the double mutant *set-25;met-2* (14), H3K23me2 is still localized in discrete foci (Figure 6F).

Collectively, our findings suggest that H3K23me2, H3K9me3 and HPL-1 define common heterochromatic sub-nuclear regions, distinct from the HPL-2 foci. Furthermore, our results indicate that the H3K23me2 pattern is not depending on HP1-like proteins and is not perturbed by the absence of H3K9 methylation.

### HPL-1 interacts with a CoREST-like complex during embryogenesis

**Figure 7.**
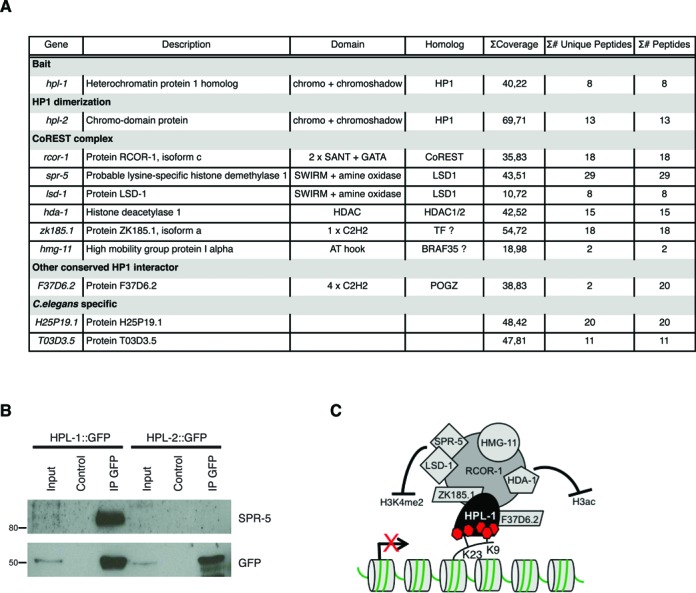
HPL-1 recruits a CoREST-like complex. (**A**) List of the HPL-1::GFP interactors, identified by MS. Gene name, description, domains, human homolog,% of sequence coverage, number of unique peptides and total number of peptides identified are shown. (**B**) HPL-1::GFP interacts with endogenous SPR-5. Total protein extracts (2 mg) from embryos of the indicated transgenic animals (*hpl-1::GFP* and *hpl-2::GFP*) were immunoprecipitated using anti-GFP antibody. The precipitates were analyzed by SDS-PAGE followed by western blotting with antibodies against GFP and SPR-5. Input = 50 μg of protein extract, Control = empty beads. (**C**) Proposed model for HPL-1 recruitment in heterochromatic foci in *C. elegans* embryos and its binding partners.

To better characterize the regions enriched for HPL-1/H3K23me2/H3K9me3, we purified HPL-1-interacting proteins from embryos. By using the stably integrated *hpl-1::GFP* transgenic strain (25), we immunoprecipitated HPL-1::GFP and we identified the interacting proteins by nano liquid chromatography—tandem mass spectrometry (nanoLC-MS/MS) analysis and sequence database searching (Figure 7A). As negative controls, HPL-1::GFP and wild-type extracts were probed with empty beads and GFP-beads, respectively. Recovered hits from the controls were subtracted from the HPL-1::GFP-IP list to generate the list shown in Figure 7A. Among the hits, we identified the REST co-repressor RCOR-1 (57), the histone deacetylase HDA-1 (58) and the histone demethylases LSD-1 and SPR-5 (59–61). Mammalian homologs of these proteins are members of the CoREST complex, required for the silencing of neuronal genes in non-neuronal cells, through chromatin remodeling (62). Zinc fingers (C2H2)-containing proteins that could work as the REST transcription factors were also identified (Figure 7A). We confirmed the specific interaction of HPL-1 with endogenous SPR-5 by co-immunoprecipitation assays, which also showed that HPL-2 does not bind to SPR-5 (Figure 7B). To further validate the interaction of HPL-1 with the CoREST complex and to explore its biological relevance, we used *C. elegans* as a genetic system. HPL-1 and HPL-2 act synergistically in vulva formation and in fertility (12). While *hpl-1* and *hpl-2* single mutants have a wild type appearance at any temperature tested, *hpl-1;hpl-2* double mutant consistently shows vulva defects, being multivulva (MUV) or presenting protruding vulva (PV), in particular at high temperature. These vulva defects are also associated to infertility at high temperature. We hypothesized that if HPL-1 and the CoREST complex act in the same pathway, CoREST components should show synergistic effects with HPL-2, but not with HPL-1. To test this hypothesis, we used an available mutant (*tm4586*) for the *rcor-1* gene, the homolog of REST corepressor 2 and 3 (RCOR2 and 3), and generated the double mutants *hpl-1;rcor-1* and *hpl-2;rcor-1*. While the double mutant *hpl-1;rcor-1* has not evident defects at any temperature tested, the double mutant *hpl-2;rcor-1* shows phenotypes that are similar to the ones presented by *hpl-1;hpl-2* double mutant at high temperature. Indeed, *hpl-2;rcor-1* lines (n = 15) show defects in vulva formation (86% of the lines show MUV and/or PV defects) (Supplementary Figure S7) and sterility (40% of the lines (n = 15) are completely sterile). The penetrance of the vulva phenotype is 12.6±7.4 (n = 137) at 15°C and increases to 41.5±1 (n = 65) at 20°C. Similarly, the sterility appears enhanced at 20°C, with 84% of the animals unable to produce viable progeny (n = 25).

These results support our hypothesis that HPL-1 and CoREST complex components interact and play a synergistic role with HPL-2 in the formation of the vulva and in fertility.

Based on our results, we propose a model (Figure 7C), in which a CoREST-like complex is recruited to specific heterochromatic regions via the direct binding of HPL-1 to H3K23me2 and H3K9me3. In the model, the recruitment of the complex would lead to gene repression or maintenance thereof through the action of H3K4me2 demethylases (LSD-1/SPR-5) and a histone deacetylase (HDA-1).

## DISCUSSION

Post-translational modifications occurring on the N-terminal tails of histone proteins contribute to the organization of eukaryotic genomes in functionally distinct nuclear domains and influence several DNA based mechanisms, like transcription and DNA replication/repair. These modifications can act alone or in combination to regulate chromatin-mediated processes (1,42,63). We selected the MS based middle-down proteomics approach to quantify single and combinatorial marks within histone H3 tails of *C. elegan*s embryos. This analysis highlighted that most of the histone H3 tails carry multiple PTMs and identified some unique features of *C. elegans* H3 PTMs, in comparison to mammals, including low levels of H3K9/K36me and high level of H3K23me in embryos. Further analyses using other stages of *C. elegans* may provide clues about the dynamics of the PTMs cross-talk during post-embryonic development. In our MS approach methylated H3K4 is barely detected, while, by ChIP studies, methylated forms of H3K4 are found consistently enriched at promoters of transcribed genes (64,65). While the antibody affinity may explain, at least in part, this discrepancy, it should be noticed that promoters of active genes represent a small percentage of the total chromatin, possibly accounting for the low level of methylated H3K4 detected by MS (39,41).

Using this approach, we identified H3K23 as a highly modified residue in *C. elegans* embryos. This was unexpected, as H3K23 is currently known to be extensively modified by acetylation, and to a much lesser extent by other PTMs such as methylation (23), ubiquitylation (66), propionylation (67) and crotonylation (2). Analyses of the interplay scores for pairs of modifications suggested that, in *C. elegans* embryos, H3K9me3 co-exists with H3K23me2 on the same histone tail. In agreement with this result, IF showed that H3K23me2 co-localizes with H3K9me3 and ChIP analysis highlighted a positive correlation between H3K23me2 and H3K9me3 and a similar chromosomal distribution of these two modified lysines along the chromosomes. The ChIP analysis also showed a positive correlation between H3K23me2 and H3K27me3 that is also supported by MS data. Indeed the co-existence frequency of K23me2:K27me3 is about 19%, but, due to the high abundance of these marks on H3 tails, their normalized frequency (interplay score) is only slightly positive (0.38).

As H3K9me3 is a repressive histone modification associated with heterochromatin and a docking site for HP1 proteins, our results suggest that H3K23me2 marks heterochromatic regions. In support of this hypothesis, H3K23me2 co-localizes with HPL-1, one of the two HP1-like proteins present in *C. elegans*. It is known that HPL-1 and HPL-2 mainly localize in distinct foci during embryogenesis (12). The identification of H3K23me2 as a mark that co-localizes with HPL-1 and H3K9me3, but not with HPL-2, not only supports the finding that HPL-1 and HPL-2 label distinct heterochromatin regions (12), but also defines these regions at higher level. Our results also suggest that H3K23me2 could act, in collaboration with H3K9me3, as a binding site for the specific recruitment of HPL-1 to discrete foci. This hypothesis is further supported by the specific and direct binding of HPL-1 to methylated forms of H3K23. However, the presence and the distribution of H3K23me2 appear completely independent of HPL proteins and H3K9me3.

IF analyses performed in embryos showed that H3K23me2 is a dynamic mark, being reduced in PGCs (P4 and Z2/Z3), in comparison to somatic cells. We attempted, without success, to identify enzymes responsible for the regulation of H3K23me2 in the PGCs by analyzing the levels of this mark in embryos extracted from available mutant alleles for putative histone methyltransferases and demethylases. We also performed RNA interference for the genes for which mutant alleles are not available, with similar results. This negative outcome can be due to redundancy. In addition, as for methylated forms of H3K4 (68), modulation of H3K23me2 in PGCs may be linked to histone replacement or to conversion to H3K23me3. Regardless of mechanism, it is of interest that this mark shows a degree of modulation in PGCs. Other histone PTMs have been reported to be reduced in Z2/Z3 during embryogenesis (48,69) and to play a relevant role in transgenerational inheritance (61,70) and future work will address the possibility that H3K23me2 might have a similar role. We found that H3K23me2 also undergoes modulation in adult germline, disappearing from nuclei in diakinesis. We noticed that in *jmjd-1.2* mutant animals, H3K23me2 reduction in oocytes is not occurring. Biochemical analysis using purified recombinant full-length JMJD-1.2 showed its ability to demethylate H3K23me2, suggesting that *jmjd-1.2* influences directly the levels of H3K23me2, specifically in the adult germline. As JMJD-1.2 is implicated in neuronal functions (32,53) and in DNA damage responses (71), it is tempting to speculate that H3K23me2 acts in these processes. However, as JMJD-1.2 is also an established histone demethylase with activity toward H3K9me2 and H3K27me2 (32,53), it is difficult at the moment to discriminate which enzymatic activity is associated to the phenotypes observed in the *jmjd-1.2* mutant. The fact that JMJD-1.2 demethylates residues associated to silent chromatin suggests that their coordinated and combined removal might be required for the regulation of heterochromatic regions.

To gain insights into the functional role of H3K23 modifications, we performed an IP/MS of HPL-1. With this approach, we identified several binding proteins, including HPL-2. The observation that HPL-1 and HPL-2 mainly do not co-localize (12) by IF suggests that their dimerization occurs outside their main respective foci. It is also possible that HPL-1 is engaged in different complexes in the embryos, one interacting with HPL-2 and another one interacting with a CoREST-like complex. Indeed, among the top hits recovered in the IP/MS, we identified homologs of members of the human CoREST complex, including RCOR-1 (CoREST 2/3 homolog), HDA-1 (HDAC1/2 homolog), LSD-1/SPR-5 (homologs of LSD1) and two uncharacterized proteins (ZK185.1 and F37D6.2) containing zinc-finger domains that may represent the homologs of REST (72) and WOC/POGZ (73,74), respectively. Furthermore, consistent with the interaction of the high mobility group (HMG) protein BRAF35 with the CoREST complex (75), our MS analysis identified the high mobility group protein HMG-11. To further validate *in vivo* the interaction between HPL-1 and the CoREST complex, we used *C. elegans* as a genetic system, testing the effect of loss of the CoREST component RCOR-1 in *hpl-1* or *hpl-2* genetic backgrounds. The data obtained indicate that loss of RCOR-1 mimics the loss of HPL-1, in a HPL-2 mutant context, further proving the interaction of HPL-1 with components of the CoREST complex and highlighting the relevance of this interaction in vital biological processes like organogenesis and fertility. Other members of the CoREST-like complex in *C. elegans* have been discovered in a suppressor screen for the postembryonic developmental defect of *sel-12*/presenilin and denominated *spr* genes (suppressor of presenilin *spr-1–5*) (59,60,76,77). In our MS analysis we recovered SPR-5, but not the other SPR proteins implicated in presenilin suppression. As we purified the complex from embryos, it is possible that, at this stage, HPL-1 interacts with a CoREST-like complex that is different from the one involved in the regulation of presenilin genes later in the development. In agreement with this, loss of *hpl-1* does not rescue *sel-12* phenotype (data not shown).

Our MS approach revealed that K23 methylation is a very abundant modification in *C. elegans* embryos. The presence of this modification as traces in mammals suggests that H3K23 methylation in the nematode could play roles that have been lost or substituted by other mechanisms during evolution. However, the fact that low-abundant methylated forms of H3K23 methylation in mammals are found to co-localize with ${\rm HP}1\alpha\beta$(78,79), suggests an evolutionary conserved function of H3K23 methylation, as also supported by its recent identification in *Tetrahymena thermophyla* (22). Interestingly, H3K23me3 was identified as a mark present in the heterochromatic and transcriptional-silent micronucleus of *Tetrahymena thermophyla*. In agreement with the localization of H3K23me3 in meiotic cells, loss of H3K23me3 was found associated to ectopic double strand breaks, thus further supporting a possible role for H3K23 methylation (at least for its tri-methylated form) in DNA damage.

Future work will be directed at understanding the biological significance of H3K23 methylation by the identification of its histone methyltransferase(s) and by the analysis of expression profiles of animals lacking *hpl-1* and subunits of the CoREST complex.

## Supplementary Material

SUPPLEMENTARY DATA
