# Supplementary material for: H3K23me2 is a new heterochromatic mark in Caenorhabditis elegans
Source: Nucleic Acids Res. 2015 Oct 17;43(20):9694–710. doi: 10.1093/nar/gkv1063 (PMC4787770; doi:10.1093/nar/gkv1063)
Supplement: SUPPLEMENTARY DATA [file supp_43_20_9694__index.html]

H3K23me2 is a new heterochromatic mark in Caenorhabditis elegans — H3K23me2 is a new heterochromatic mark in Caenorhabditis elegans — SUPPLEMENTARY DATA 

# H3K23me2 is a new heterochromatic mark in *Caenorhabditis elegans*

## SUPPLEMENTARY DATA

- SUPPLEMENTARY DATA
- SUPPLEMENTARY DATA
- SUPPLEMENTARY DATA
- SUPPLEMENTARY DATA
- SUPPLEMENTARY DATA
- SUPPLEMENTARY DATA
- SUPPLEMENTARY DATA
- SUPPLEMENTARY DATA
- SUPPLEMENTARY DATA
- SUPPLEMENTARY DATA
