# Supplementary material for: H3K23me2 is a new heterochromatic mark in Caenorhabditis elegans
Source: Nucleic Acids Res. 2015 Oct 17;43(20):9694–710. doi: 10.1093/nar/gkv1063 (PMC4787770; doi:10.1093/nar/gkv1063)

## SUPPORTING INFORMATION

### Supporting methods

### Dot Blot

Synthetic biotinylated peptides of histone H3 lysine 23 unmodified, mono-, di- and tri-methylated (H3K23me0, H3K23me1, H3K23me2 and H3K23me3) (JPT Peptide Technologies) were spotted in dilution series (ranging from 100 to 10 ng) on Nitrocellulose membrane (Hybond-C Extra; Amersham Biosciences). The membrane was blocked for 1 hour in 5% non-fat milk dissolved in PBS-T (PBS (pH 7.2), 0.01% Tween-20) and the following antibodies were tested for specificity with a 1:2000 dilution in the blocking solution: Streptavidin (SouthernBiotech 7100-05), H3K23me1 (Active Motif 39387); H3K23me2 (Active Motif 39653), H3K23me3 (Active Motif 61499). After incubation with primary antibodies, the membrane was washed four times and probed with secondary anti-rabbit HRP antibody (Vector laboratories). After three washes in PBS-T, the signal was detected by enhanced chemiluminiscence (SuperSignal West Pico Chemiluminescent Substrate, Thermo scientific).

### Peptide competition assay

Antibodies against H3K23me1, me2 and me3 diluted at 1:2000 in PBS, 0,10% tween, 5% milk were preincubated with 2 mg/ml of indicated peptides (H3 11-30 K23me0, me1, me2 and me3) before immunoblotting. Between 1 and 15mg of total protein extracts from embryos were loaded on the gel. Antibody against histone H3 (ab1791, Abcam) was used as loading control.

**Figure S1. PTMs distribution on histone H3 tails.** (a) Relative abundance of single PTMs on histone H3 tails sorted by decreasing values. (b) Quantification of histone H3 tails grouped by number of PTMs. Doubly modified histone tails represent more than half of the entire dataset. (c) Violin plot of interplay value distribution. The median (-2.38) is below 0, indicating that PTMs tend to be mutually exclusive more frequently than co-dependent.

**Figure S2**. **Validation of antibodies against methylated H3K23.** (a) Immuno–dot blot of the 3 indicated antibodies. 10, 50 and 100 ng of the indicated linear H3 biotinylated peptides were spotted on the membrane. Streptavidin blot is loading control. (b) Peptide competition assay of methylated H3K23 antibodies. Western blot analysis of protein extracts from embryos with methylated H3K23 antibodies, pre-adsorbed with 2mg/ml of indicated peptides. H3 was used as loading control. H3 peptides (11-30) were either unmodified (me0) or methyl ated (me1, me2 or me3) at position K23 of H3. Note that antibodies against H3K23me1 and me3 show a certain degree of cross-reactivity with H3K23me2 peptides, while antibody against H3K23me2, mainly used in this study, appears specific.

**Figure S3. H3K23me2/3 in PGCs.** Embryos were stained by the indicated antibodies: H3K23me2/3 (red), PGL-1 (green). PGL-1 antibody stains specifically PGC cells. DNA was counterstained by DAPI (Blue). Scale bar: 5 μm

**Figure S4. Purified JMJD-1.2 and Co-localization experiments for H3K23me2 in embryos.** (a) Purified Flag-tagged recombinant JMJD-1.2 expressed in insect cells. Eluted fractions were subjected to SDS-PAGE and stained with Comassie blue. Marker is molecular weight standard. Arrowhead indicates the JMJD-1.2 purified (b) Representative images of N2 embryos fixed and stained with indicated antibodies: H3K23me2 (red), H3K4me2, H3K27me3 or H3K9me2 (green). Overlay is shown on the right. Scale bars: 5 μm.

**Figure S5. Selected regions for H3K23me2 ChIP-qPCR**. (a) Screenshots of modEncode genome browser from each of the 6 chromosomes, windows of 20kbp. ChIP-seq tracks for H3K4me3 and H3K9me3 in N2 early embryos are shown. Regions in which primers for qPCR have been designed are indicated as "chromosome number" followed by the lysine residue of interest (K4 or K9). (b) Sequences of the primers used for H3K23me2 ChIP-qPCR.

**Figure S6. Characterization of HPL proteins.** (a) Embryos from animals carrying HPL-1::GFP transgene, fixed and stained with H3K9me3 (red) and GFP (green) antibodies. DNA is counterstained with DAPI (blue). Representative image of a single

nucleus is shown. Scale bar: 2μm. (b) Embryos from transgenic animals carrying *hpl-1::GFP* or *hpl-2::GFP* were fixed and stained with H3K23me2 (red) and GFP (green) antibodies. Representative images of several embryonic nuclei are shown. Scale bar: 5 μm (c) SDS-PAGE analysis of purified GST-tagged recombinant HPL-1 and HPL-2a expressed in bacteria. Marker is a molecular weight standard. Proteins were stained using Coomassie blue.

**Supplementary Figure 7. Genetic interaction of CoREST member rcor-1 with HPL proteins.** Differential Interference Contrast (DIC) micrographs of the indicated strains. Ventral view of adult animals. Single mutant animals for *hpl-1, hpl-2, rcor-1* and double mutant animals for *hlp-1;rcor-1* show wild type vulva (arrow). Several embryos (asterisks) are also evident in the uterus, indicative of fertility. Double mutant for *hpl-2;rcor-1* and *hpl-1;hpl-2* are multivulva (arrowhead) and show reduced body size, reduced/absent germline and not embryos in the uterus. Scale bar: 20μm.

**Supplementary Table 1. MS results from the middle-down analysis of histone H3 N-terminal tails.** The first sheet of the table is divided into four categories; (i) 'Combinatorial codes' represents the different peptides identified and quantified from the MS analysis (average of two biological replicates). The relative quantification of the various modified peptides is estimated considering the total sum as 100%. The following columns (ii to iv) are deconvoluted data obtained from this initial peptide list. (ii) 'Single marks frequency" is the deconvoluted relative abundance of the individual PTMs. The value was obtained by summing all peptides in the column 'Combinatorial codes' containing the given mark. (iii) 'Binary marks frequency' is the calculated relative abundance of binary marks, quantified using the same approach as the single marks. (iv) 'Binary marks normalized frequency (interplay)' is the interplay score between binary marks, calculated as described in the main text. This value represents the interdependency of two PTMs, and it was used to estimate which marks are more likely to co-exist or be mutually exclusive. The second sheet includes the relative abundance of the quantified peptides in each of the four LC-MS replicates, including two technical replicates for each of the biological replicate.

**Supplementary Table 2. Lists of putative histone methyltransferases (KTMs) and demethylases (KDMs) analyzed by immunofluorescence.** Gene name, WormBase ID and mutant alleles used are indicated. In absence of mutant alleles, RNA interference, by feeding protocol, was used to downregulate the target gene. Note that the class of histone demethylase includes genes coding for JmjC-containing proteins.

**Supplementary Figure 1**

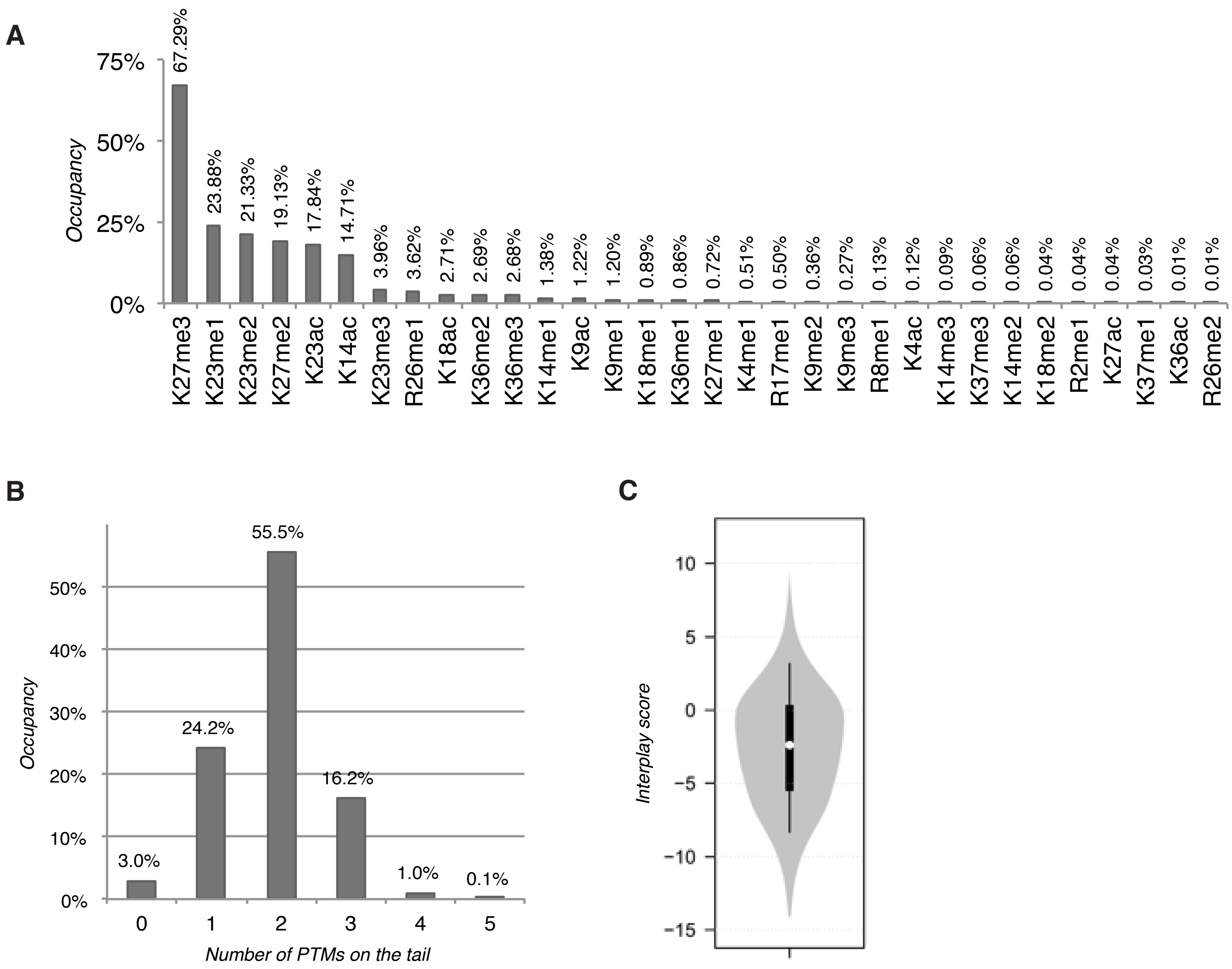

## Supplementary Figure 2

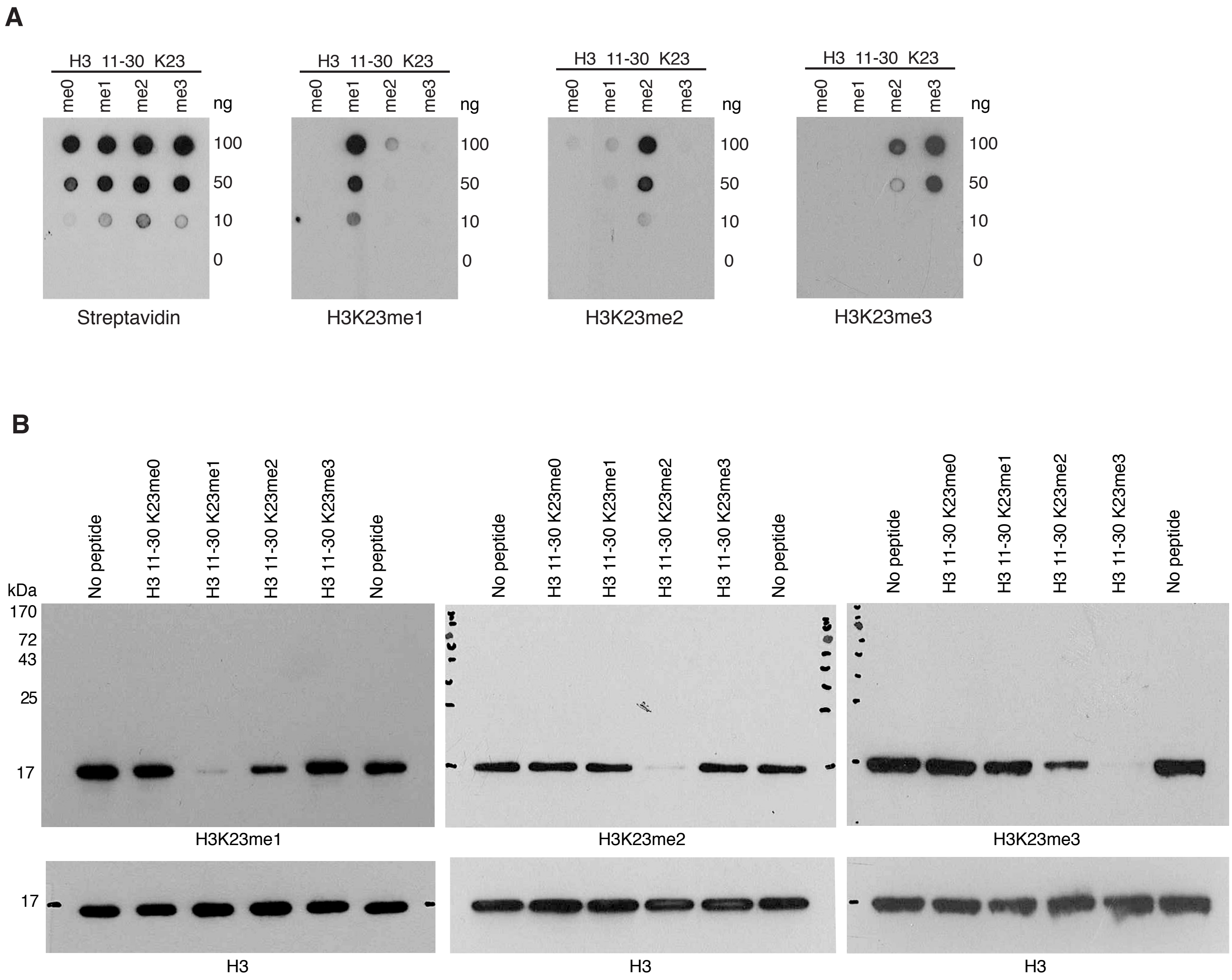

**Supplementary Figure 3**

H3K23me2/PGL-1 H3K23me3/PGL-1

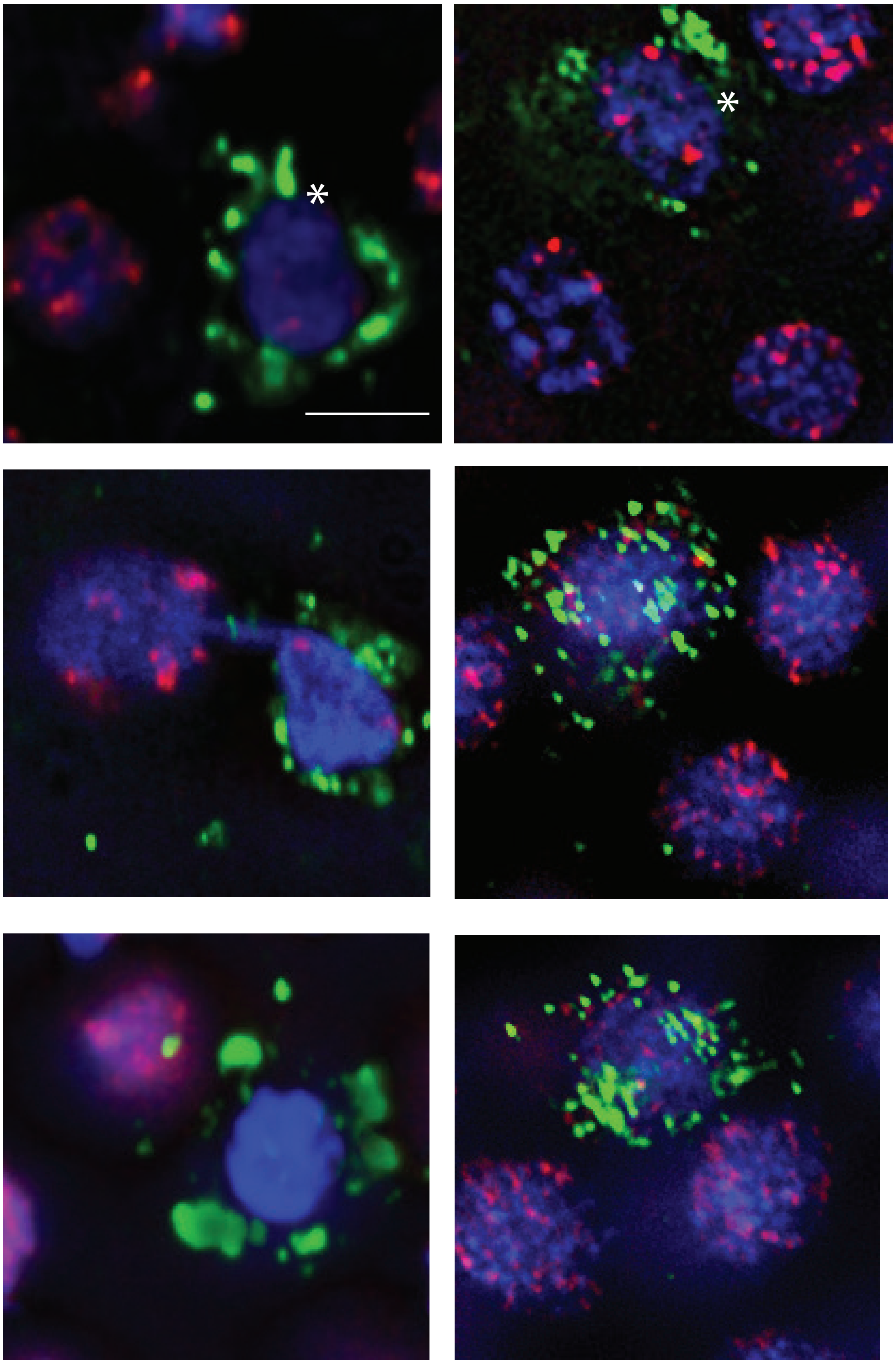

**A**

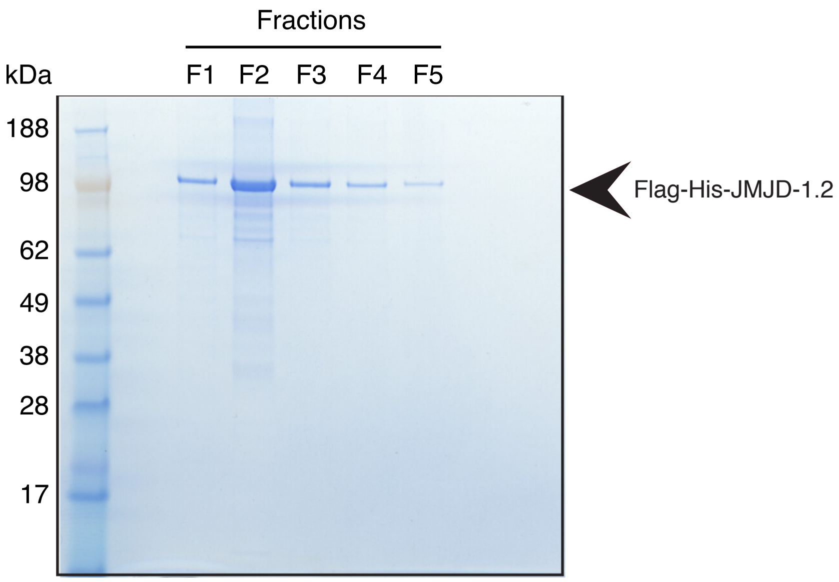
Fractions
kDa
F1
F2
F3
F4
F5
188
98
62
49
38
28
17
Flag-His-JMJD-1.2

**B**

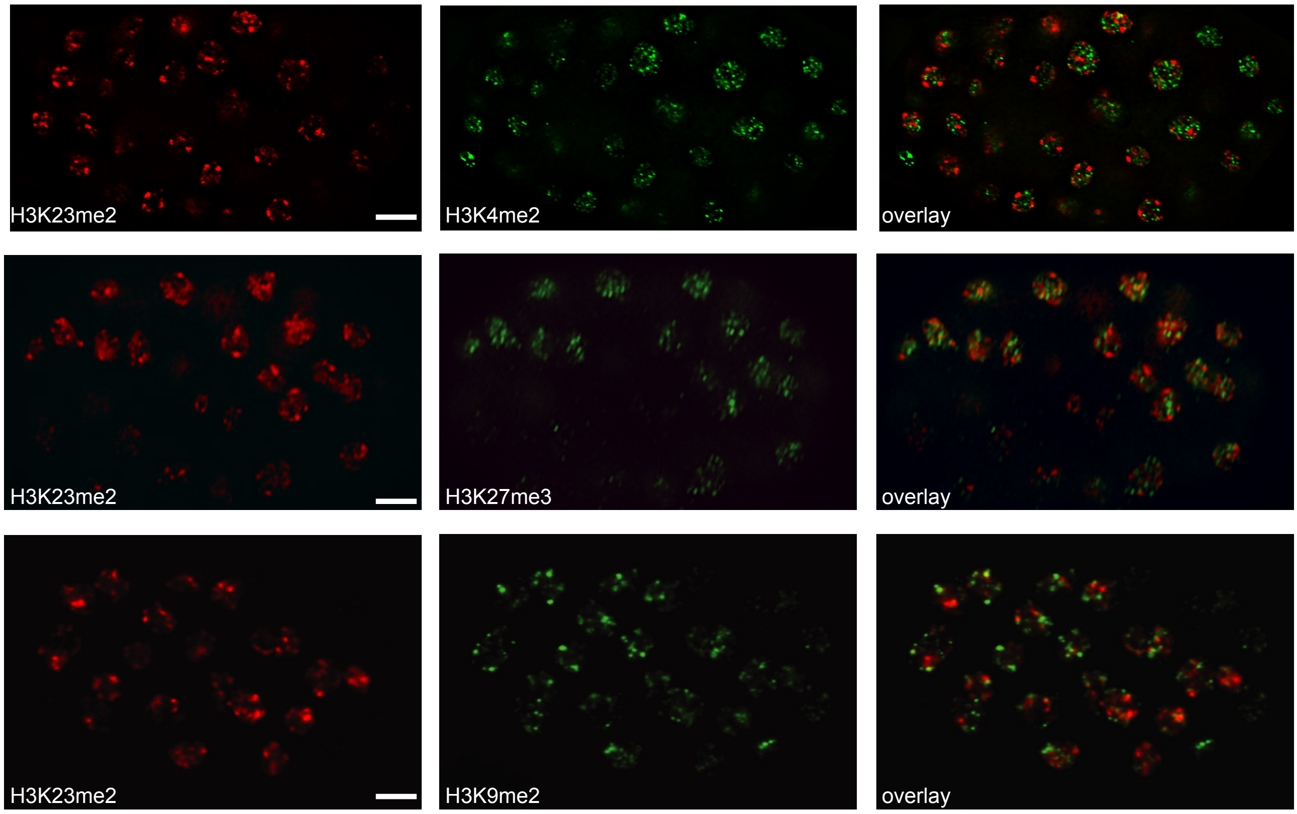
H3K23me2
H3K4me2
overlay
H3K23me2
H3K27me3
overlay
H3K23me2
H3K9me2
overlay

**A**

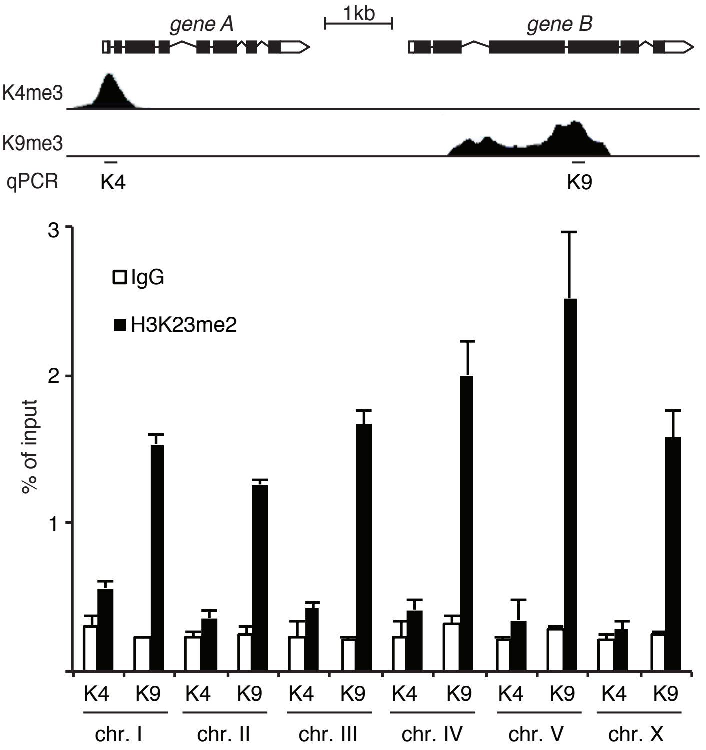


**B**

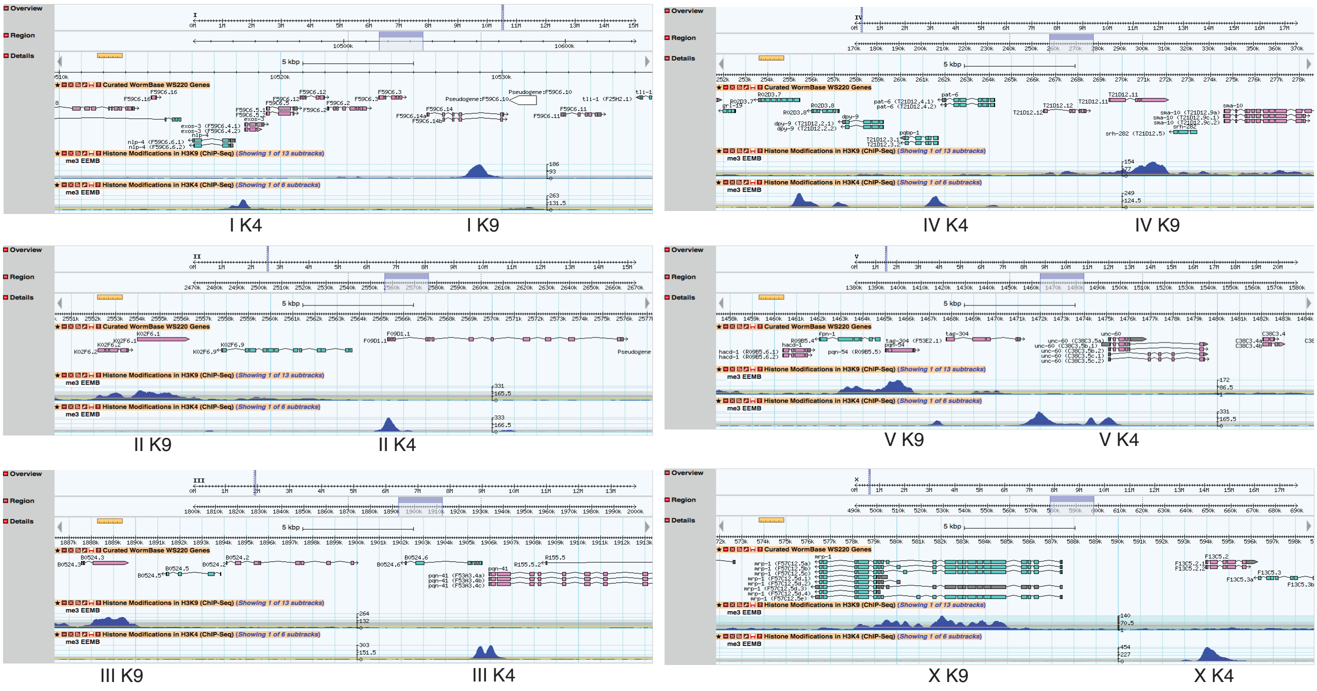


**C**

| name | sequence | name | sequence | name | sequence | name | sequence |
|---|---|---|---|---|---|---|---|
| I_K4_fw | acgttattcctcctgcggta | I_K4_rv | gaaagctccaggttgagtgg | I_K9_fw | acgacgttgaaaccaggaac | I_K9_rv | aaccgttaagcatggaggtg |
| II_K4_fw | acgacaaaagtccgcaagat | II_K4_rv | gttccaccttttcgggagat | II_K9_fw | gagcctctggagcgtctatg | II_K9_rv | tgcatcaaataacgctttgc |
| III_K4_fw | ccattccaaagctgctcaat | III_K4_rv | gattctggatcggtttcacc | III_K9_fw | gaaacctcagcagggtgcta | III_K9_rv | gccaggacactttttccaga |
| IV_K4_fw | cttcgcaagtccctcatcat | IV_K4_rv | gagcacctggttgtccaaat | IV_K9_fw | ggtgaacctgccagtacgac | IV_K9_rv | tgtcgtgctgatttttgctt |
| V_K4_fw | tgattctatttccccaccaca | V_K4_rv | gaccatgacaccggaactct | V_K9_fw | tccaaaccactcaatgtgct | V_K9_rv | aacacaggtggattggcact |
| X_K4_fw | aagagcgatctcaccggata | X_K4_rv | gaccagatggggtccttttt | X_K9_fw | ttcttccaagtggggttacg | X_K9_rv | cgaaatggaagccataaagc |

A

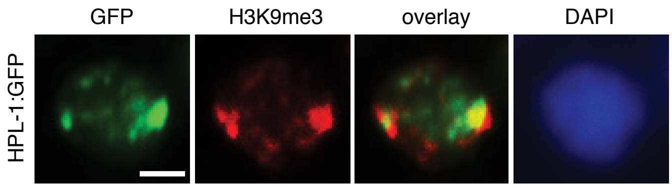
GFP
H3K9me3
overlay
DAPI
HPL-1:GFP

B

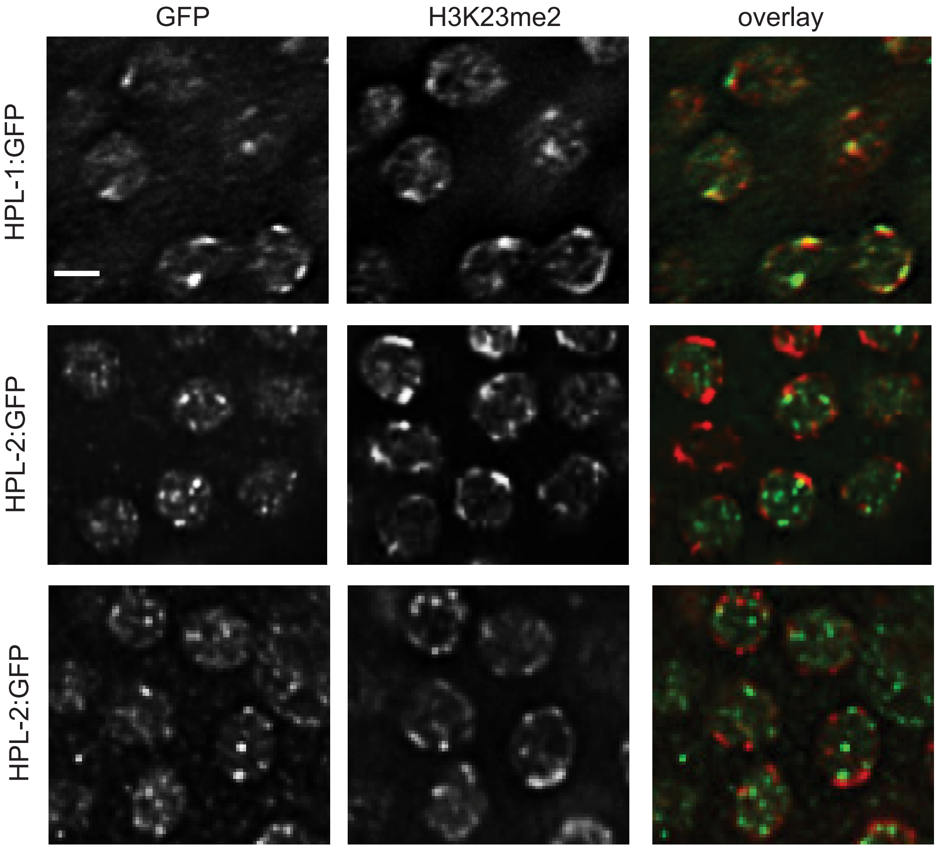
GFP
H3K23me2
overlay
HPL-1:GFP
HPL-2:GFP
HPL-2:GFP

C

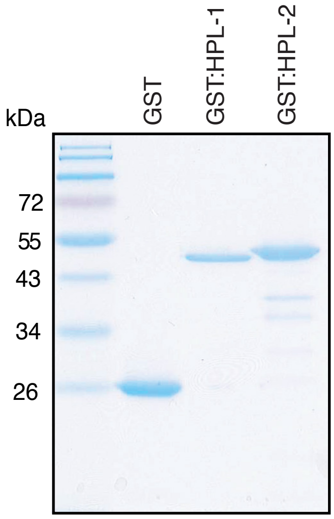
kDa
GST
GST:HPL-1
GST:HPL-2
72
55
43
34
26

**Supplementary Figure 7**

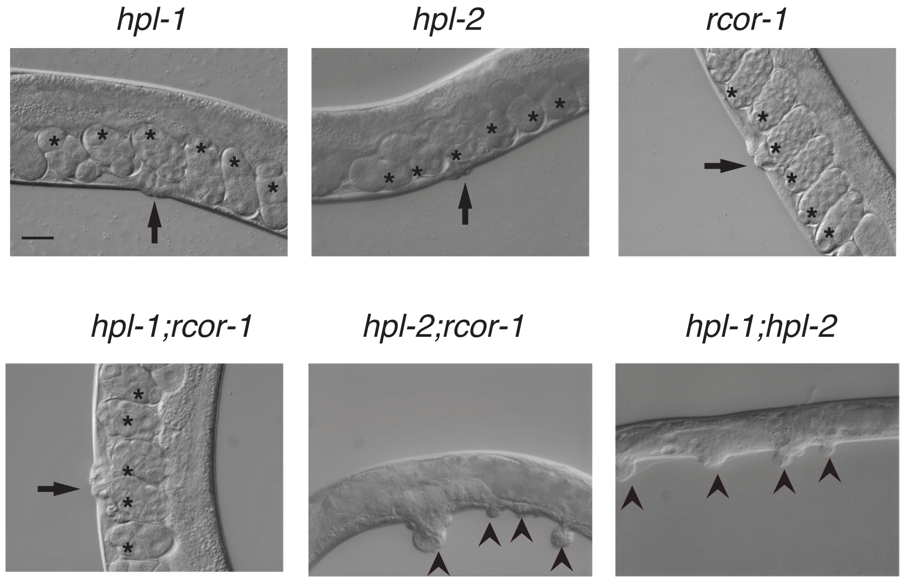

Supplement: SUPPLEMENTARY DATA [file supp_gkv1063_nar-01499-m-2015-File002.pdf]
