# Supplementary figures and images for: H3K23me2 is a new heterochromatic mark in Caenorhabditis elegans
Source: Nucleic Acids Res. 2015 Oct 17;43(20):9694–710. doi: 10.1093/nar/gkv1063 (PMC4787770; doi:10.1093/nar/gkv1063)

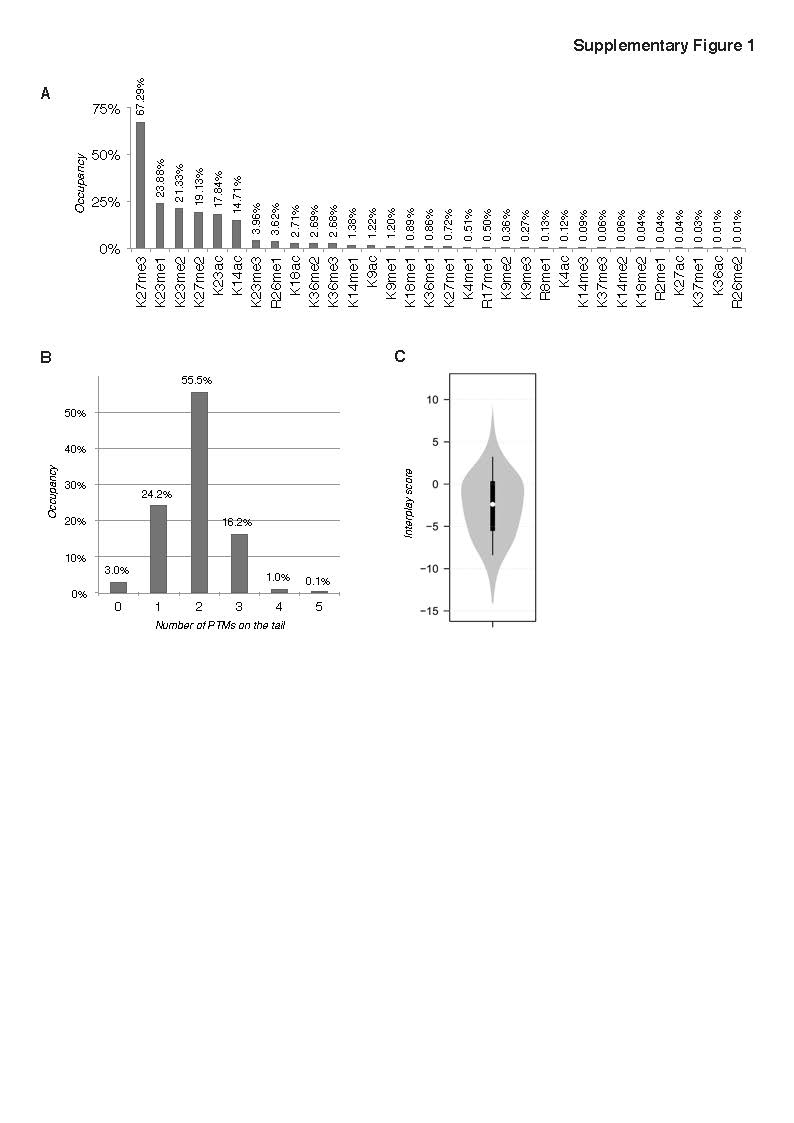

Supplement: SUPPLEMENTARY DATA [file supp_gkv1063_nar-01499-m-2015-File003.jpg]

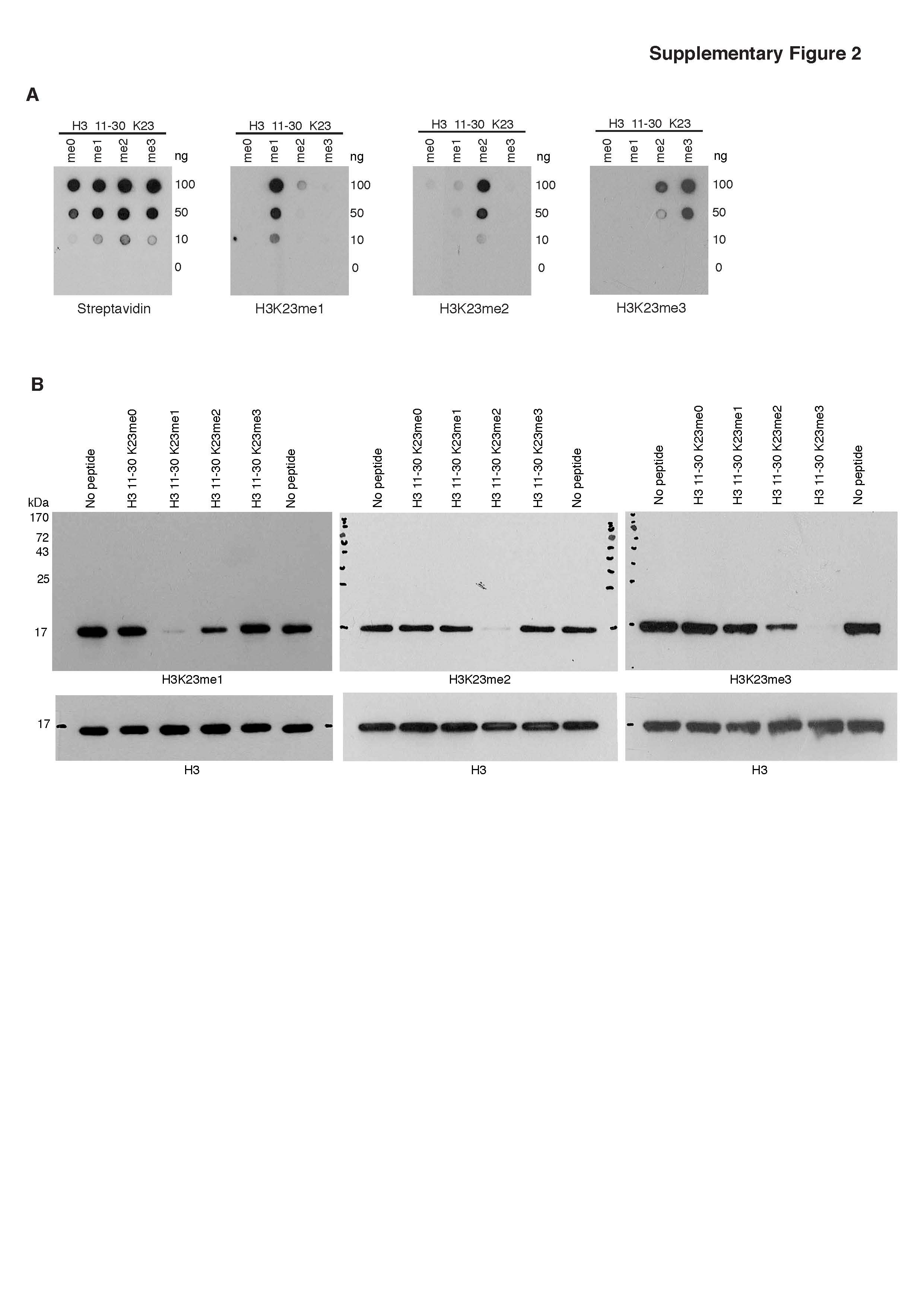

Supplement: SUPPLEMENTARY DATA [file supp_gkv1063_nar-01499-m-2015-File004.jpg]

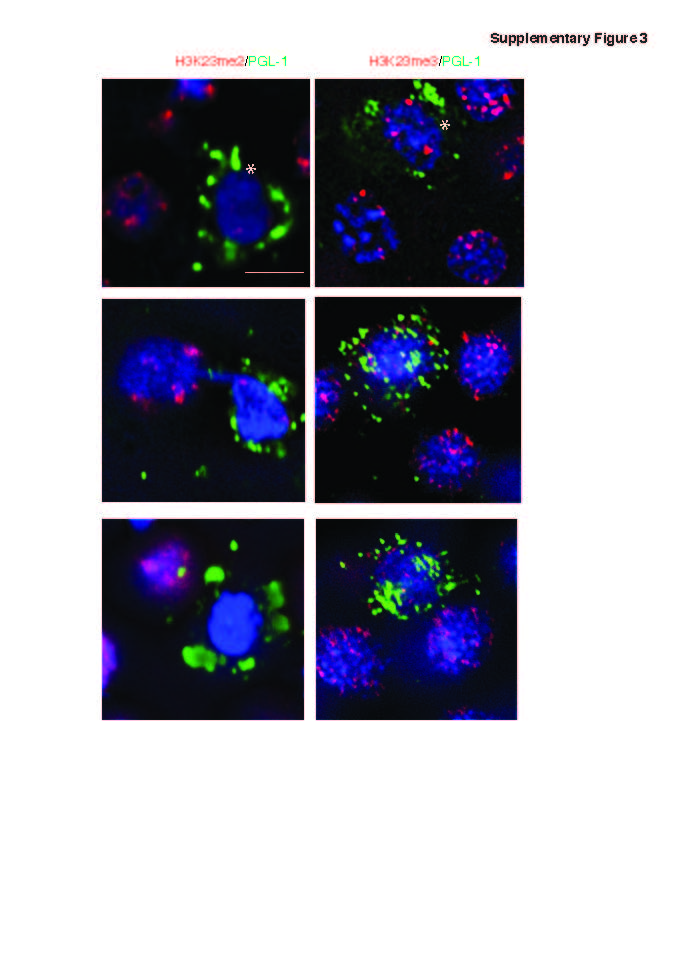

Supplement: SUPPLEMENTARY DATA [file supp_gkv1063_nar-01499-m-2015-File005.jpg]

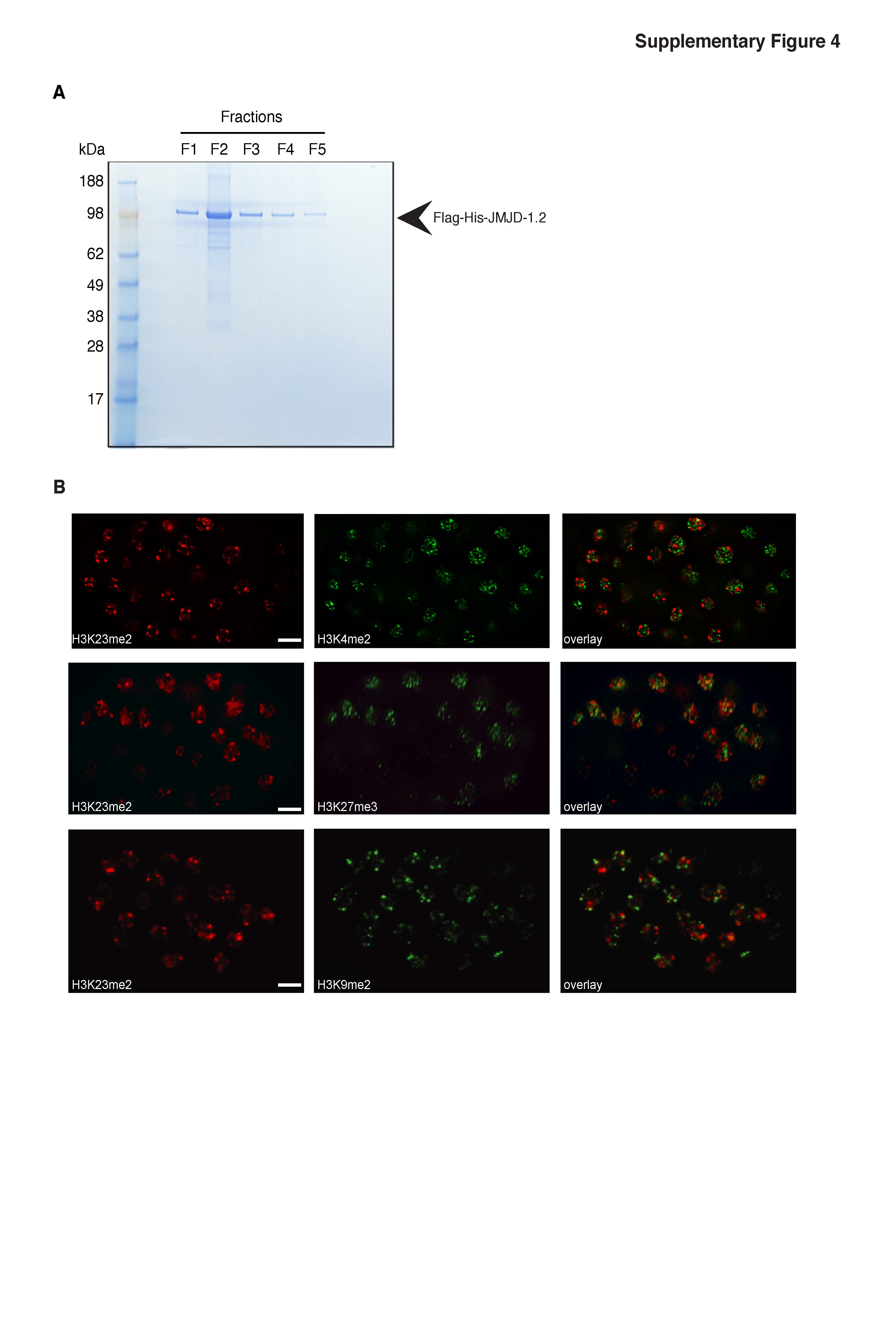

Supplement: SUPPLEMENTARY DATA [file supp_gkv1063_nar-01499-m-2015-File006.jpg]

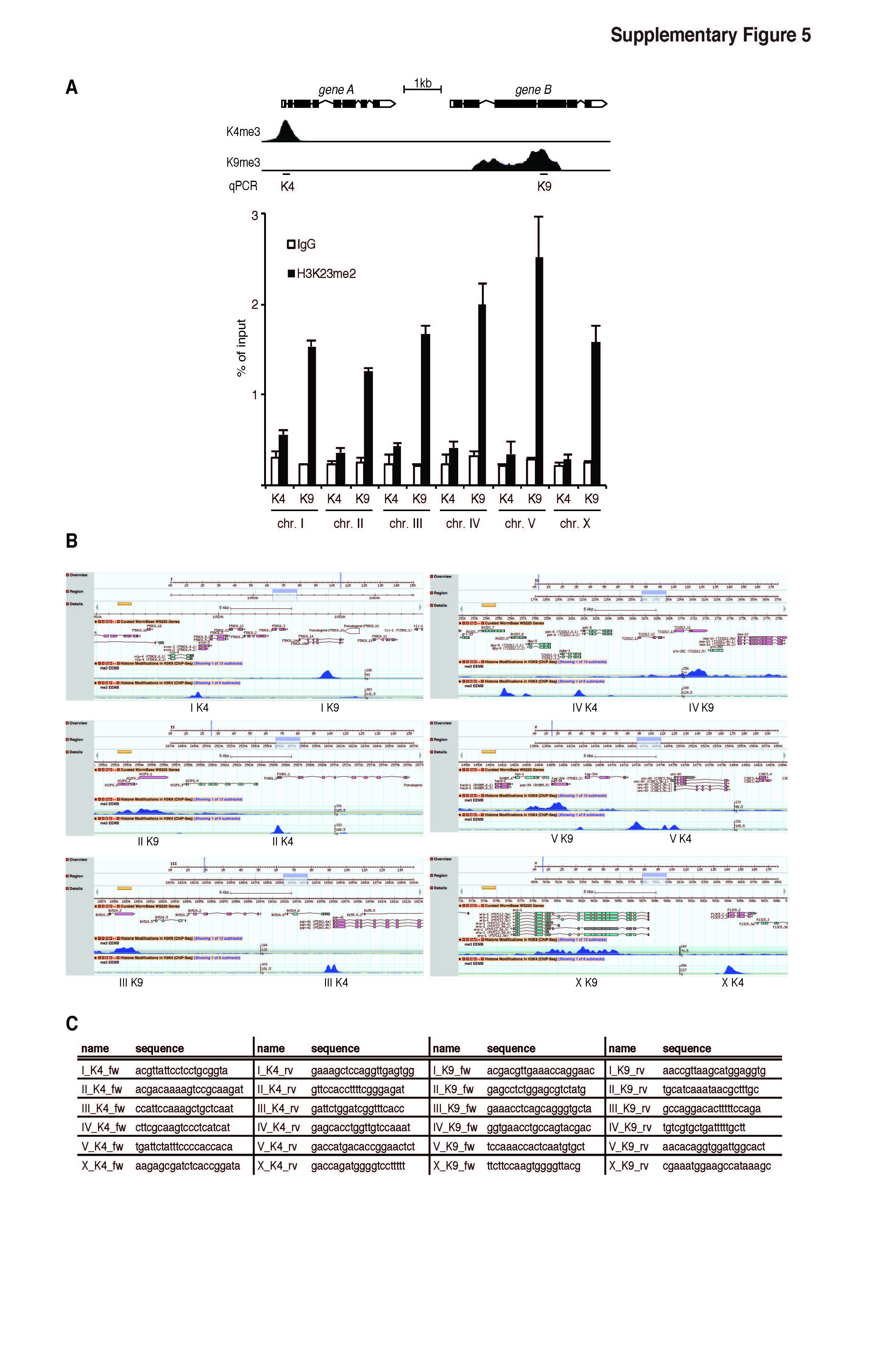

Supplement: SUPPLEMENTARY DATA [file supp_gkv1063_nar-01499-m-2015-File007.jpg]

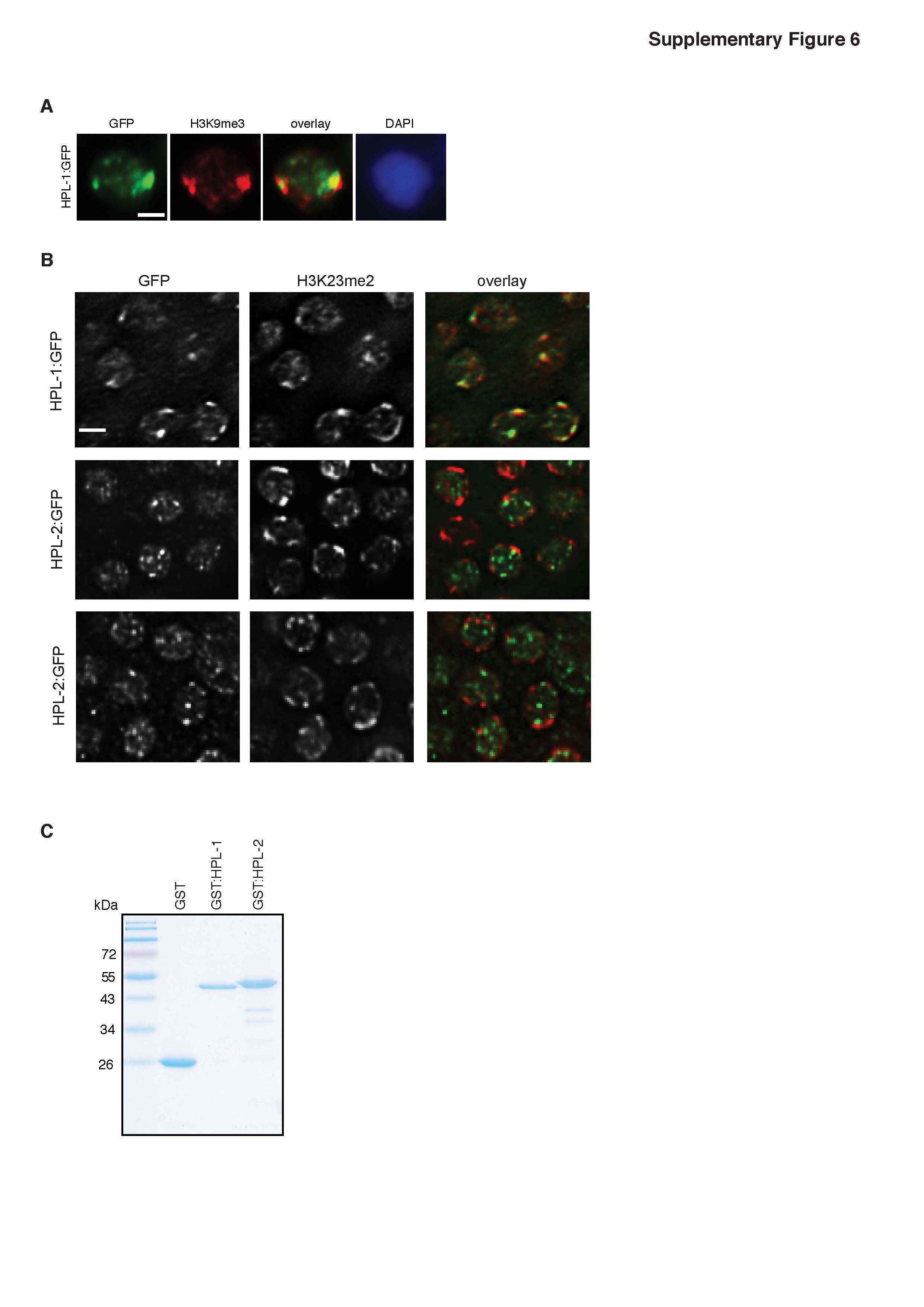

Supplement: SUPPLEMENTARY DATA [file supp_gkv1063_nar-01499-m-2015-File008.jpg]

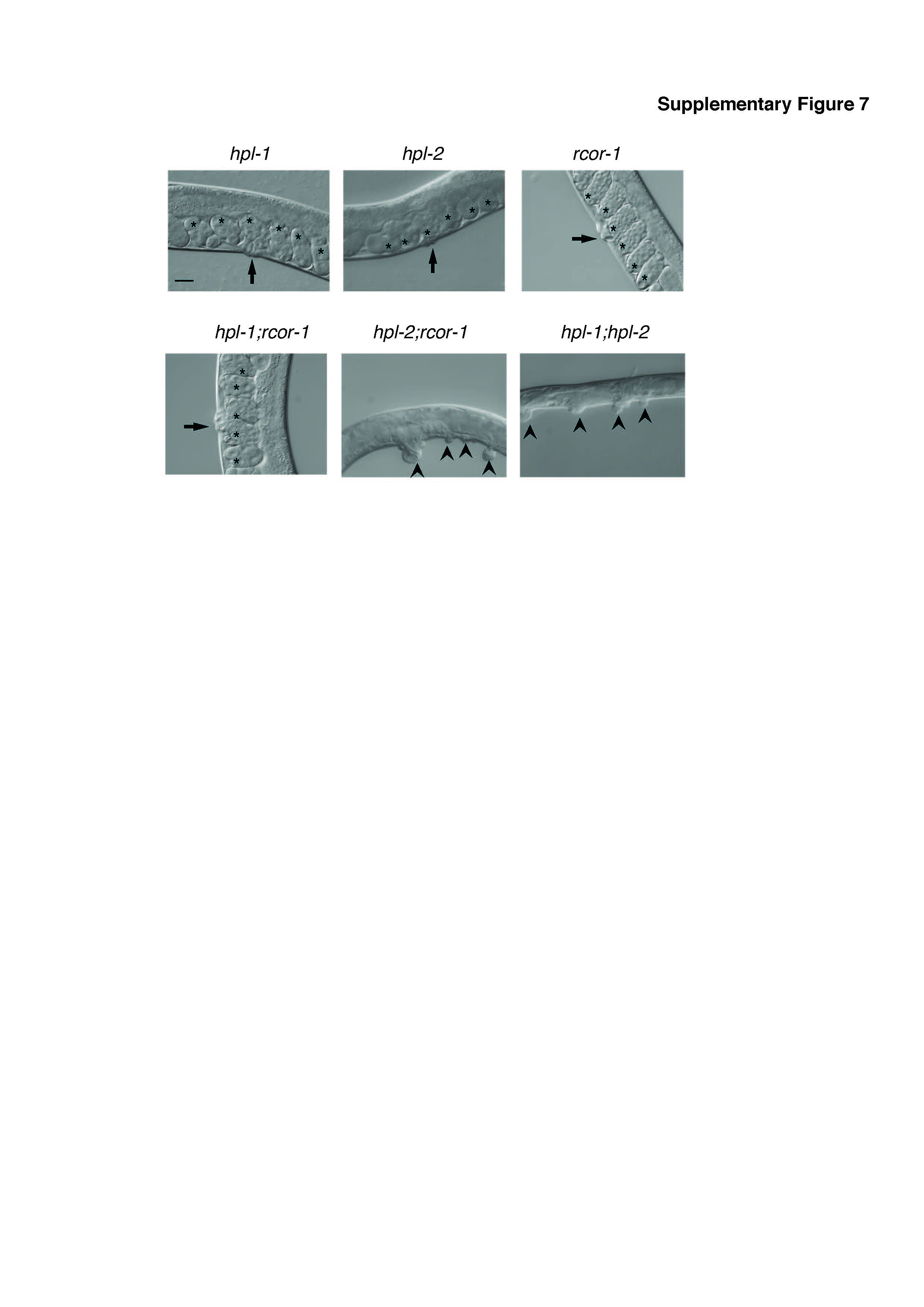

Supplement: SUPPLEMENTARY DATA [file supp_gkv1063_nar-01499-m-2015-File009.jpg]
